# Hyperbaric Oxygen Boosts PD‐1 Antibody Delivery and T Cell Infiltration for Augmented Immune Responses Against Solid Tumors

**DOI:** 10.1002/advs.202100233

**Published:** 2021-06-03

**Authors:** Xin Liu, Ningbing Ye, Sha Liu, Jiankun Guan, Qingyuan Deng, Zhijie Zhang, Chen Xiao, Ze‐yang Ding, Bi‐xiang Zhang, Xiao‐ping Chen, Zifu Li, Xiangliang Yang

**Affiliations:** ^1^ National Engineering Research Center for Nanomedicine College of Life Science and Technology Huazhong University of Science and Technology Wuhan 430074 P. R. China; ^2^ Hepatic Surgery Center and Hubei Key Laboratory of Hepatic‐Biliary‐Pancreatic Diseases National Medical Center for Major Public Events Tongji Hospital Tongji Medical College Huazhong University of Science and Technology Wuhan 430030 P. R. China; ^3^ Key Laboratory of Molecular Biophysics of Ministry of Education College of Life Science and Technology Huazhong University of Science and Technology Wuhan 430074 P. R. China; ^4^ Hubei Key Laboratory of Bioinorganic Chemistry and Materia Medical Huazhong University of Science and Technology Wuhan 430074 P. R. China; ^5^ Wuhan Institute of Biotechnology High Tech Road 666 East Lake High Tech Zone Wuhan 430040 P. R. China; ^6^ GBA Research Innovation Institute for Nanotechnology Guangdong 510530 P. R. China

**Keywords:** extracellular matrix, hyperbaric oxygen therapy, immunotherapy, PD‐1 antibody, solid tumors

## Abstract

Aberrant mechanical properties and immunosuppression are the two key factors that limit the antitumor efficacy of T cell immune checkpoint blockade inhibitors, e.g., programmed cell death‐1 antibody (PD‐1 Ab), against solid tumors in the clinic. This study leverages hyperbaric oxygen (HBO) for the first time to address these two issues and reports the PD‐1‐Ab‐mediated immune responses against various stroma‐rich solid malignancies. The results demonstrate that HBO promoted PD‐1 Ab delivery and T cells infiltration into tumor parenchyma by depleting the extracellular matrix's main components, such as collagen and fibronectin. Furthermore, HBO disrupts hypoxia‐mediated immunosuppression and helps PD‐1 Ab trigger robust cytotoxic T lymphocytes and long‐lasting immunological memory to inhibit tumor relapses. Such enhanced immune responses are effective in solid tumors from rodents and the cancer cells from hepatocellular carcinoma patients. The results illustrate that HBO bolsters antitumor efficacy of PD‐1 Ab, and the HBO–PD‐1 Ab combination is a promising stroma‐rich solid tumors’ treatment in the clinic.

## Graphical abstract

It is revealed that hyperbaric oxygen (HBO) depleted extracellular matrix's major components, including collagen and fibronectin, thereby promoting the delivery of PD‐1 Ab and the infiltration of T cell into tumor parenchyma. Furthermore, HBO disrupted hypoxia‐mediated immunosuppression and helped PD‐1 Ab trigger robust cytotoxic T lymphocytes and long‐lasting immunological memory effect for the inhibition of tumor relapses.

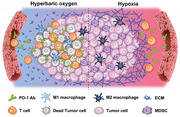

## 1 Introduction

T cell immune checkpoint blockade (ICB) inhibitors, e.g., programmed cell death‐1 antibody (PD‐1 Ab), have achieved remarkable success in some cancers’ treatment;^[^ ^1^ ^]^ however, their efficacy against stroma‐rich solid malignancies is modest.^[^ ^2^ ^]^ The ICB antibodies and cytotoxic T lymphocytes (CTLs) should reach the vicinity of cancer cells to initiate the potent T‐cell‐mediated immune responses and tumor‐cell eradication.^[^ ^3^ ^]^ However, for desmoplastic solid tumors,^[^ ^4^ ^]^ such as hepatocellular carcinoma (HCC),^[^ ^5^ ^]^ pancreatic ductal adenocarcinoma (PDAC),^[^ ^6^ ^]^ and triple‐negative breast cancer (TNBC),^[^ ^7^ ^]^ it is difficult for even small molecule‐based antitumor drugs to penetrate through the “rampart” dense extracellular matrix (ECM),^[^ ^8^ ^]^ not to mention the delivery of nanometer‐sized PD‐1 Ab^[^ ^9^ ^]^ or the infiltration of micrometer‐scale CTLs.^[^ ^10^ ^]^ Therefore, normalizing aberrant mechanical tumor microenvironment (TME) in desmoplastic tumors can considerably enhance the antitumor efficacy of ICB antibodies‐based immunotherapy.^[^ ^11^ ^]^ To this end, numerous approaches have been proposed to deplete the excessive ECM surrounding tumor nests for augmented immunotherapy.^[^ ^7^, ^12^, ^13^ ^]^

Hypoxia and hypoxia‐mediated immunosuppression are known to compromise the antitumor efficacy of ICB inhibitors in solid tumors.^[^ ^14^ ^]^ Most solid tumors are in a severe hypoxic condition because of an imbalance between the cancer cell's fast‐growing‐induced oxygen consumption and the inadequate oxygen supply by the chaotic tumor blood vessels.^[^ ^15^ ^]^ By activating the hypoxia‐inducible factor‐1*α* (HIF‐1*α*) signaling pathway, hypoxia upregulates the collagen genes, prolyl‐4‐hydroxylase *α*‐subunit, and connective tissue growth factor (CTGF), thereby promoting collagen synthesis and dense ECM formation.^[^ ^16^ ^]^ Besides the physical exclusion of ICB antibodies and CTLs, hypoxia impacts the functions of CTLs by fostering a suppressive immune microenvironment via multiple pathways.^[^ ^14^, ^15^ ^]^ Hypoxia directly upregulates programmed cell death‐ligand 1 (PD‐L1) expression to exhaust and suppress CTLs.^[^ ^17^ ^]^ The hypoxia/HIF‐1*α*/Adenosine signaling axis facilitates recruitment and accumulation of immunosuppressive regulatory T (T_reg_) cells in solid tumors.^[^ ^18^ ^]^ Likewise, myeloid‐derived suppressive cells (MDSCs) and M2 phenotype tumor‐associated macrophages (TAMs) are formed from myeloid cells or monocytes under hypoxia.^[^ ^19^ ^]^ These suppressive immune cells further secrete inhibitory cytokines, such as transforming growth factor‐*β* (TGF‐*β*) and interleukin‐10 (IL‐10) that together orchestrate a suppressive immune microenvironment.^[^ ^20^ ^]^ Thus, it is of crucial significance to unleash the ICB antibody‐mediated immunotherapy by overcoming hypoxia in solid tumors.^[^ ^21^ ^]^ Diverse strategies, such as respiratory hyperoxia,^[^ ^18^ ^]^ delivering oxygen via nanocarriers,^[^ ^22^, ^23^, ^24^ ^]^ in situ oxygen generation through chemical reactions,^[^ ^25^ ^]^ blood vasculature normalization to enhance blood perfusion and oxygenation,^[^ ^26^ ^]^ decreasing oxygen consumption,^[^ ^27^ ^]^ and utilizing hypoxia‐activated prodrug TH‐302,^[^ ^19^ ^]^ have been used to overcome tumor hypoxia and invigorate CTLs in the hostile TME of solid tumors. However, most of these studies are performed in preclinical models and have a considerable long path to bedside applications.

**Figure 1:**
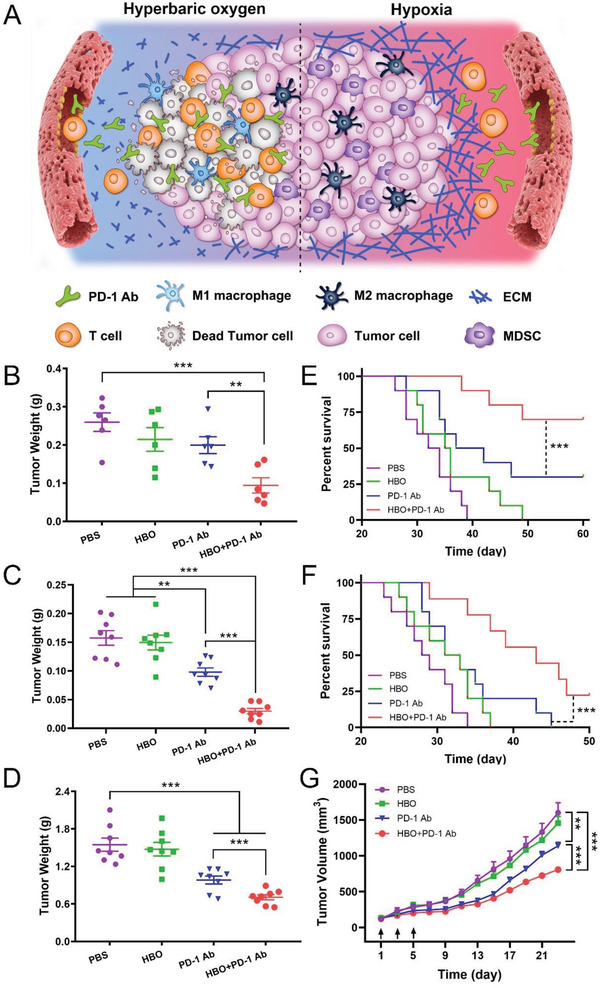
HBO boosts antitumor effect of PD‐1 Ab in stroma‐rich solid tumors. A) Schematic illustration of amplified PD‐1‐Ab‐mediated immune responses with HBO. B) Tumor weight (*n* = 6) and E) survival curve (*n* = 10) of H22 orthotopic tumor‐bearing BALB/c mice after different treatments. C) Tumor weight (*n* = 8) and (F) survival curve (*n* = 10) of Panc02 orthotopic tumor‐bearing BALB/c mice after different treatments. D) Tumor weight (*n* = 8) and (G) tumor growth curves of 4T1 orthotopic tumor‐bearing BALB/c mice after different treatments (*n* = 8). Black arrows represent treatments. Error bars indicate SEM. Statistical significance was calculated by *t*‐test. *P*‐values: *, *P* < 0.05; **, *P* < 0.01; ***, *P* < 0.001.

Hyperbaric oxygen (HBO) therapy is one of the most efficient means of conquering hypoxia within solid tumors.^[^ ^28^ ^]^ The HBO therapy delivers oxygen independent of hemoglobin in the blood, which is an incomparable advantage over other oxygen delivery protocols.^[^ ^29^ ^]^ Our earlier study utilized HBO to overcome tumor hypoxia and enhance chemotherapy of Doxil in rodent HCC H22 and Bel7402 models.^[^ ^30^ ^]^ The significantly increased reactive oxygen species (ROS) concentration plays a vital role during the HBO treatment; our study synergized HBO with upconversion nanophotosensitizers for enhanced photodynamic cancer therapy against the rodent TNBC 4T1 model.^[^ ^31^ ^]^ In this study, we used HBO to augment PD‐1 Ab‐mediated immunotherapy against a wide spectrum of stroma‐rich solid tumors, including HCC, PDAC, and TNBC. By depleting ECM and overcoming hypoxia, HBO promotes the PD‐1 Ab delivery, enhances CTLs infiltration, suppresses MDSCs recruitment, and the formation of M2 phenotype TAMs, thereby reprogramming TME from being immunosuppressive to immunostimulatory (**Figure** **1**A). Our results suggest that HBO empowers PD‐1 Ab for stroma‐rich solid tumors treatment.

## 2 Results

### 2.1 HBO Enhances Antitumor Efficacy of PD‐1 Ab

Three stroma‐rich rodent solid tumors,^[^ ^4^ ^]^ HCC H22, PDAC Panc02, and TNBC 4T1, were selected for this study. All three desmoplastic tumor tissues exhibited high expression of PD‐L1 (Figure S1, Supporting Information). We chose these models to ensure a desirable curative effect with PD‐1 Ab. Figure 1B–G shows PD‐1 Ab achieving a modest control on tumor growth in all tumors and slightly extending the survival time of mice with orthotopic tumors of H22 and Panc02. However, HBO exerted a negligible effect on inhibiting tumor growth, which was different from the results obtained from the lung cancer‐bearing mice using respiratory hyperoxia.^[^ ^18^ ^]^ This difference could be most likely attributed to employing different cancer models or specific treatment protocols. HBO enhances the antitumor effects of PD‐1 Ab in all tumors (Figure 1B–D; and Figures S2–S10, Supporting Information). The survival median was prolonged from 40 days for PD‐1 Ab to more than 60 days for the combination of HBO and PD‐1 Ab (HBO+PD‐1 Ab) in H22 orthotopic tumor (Figure 1E) and from 32 to 43 days in the Panc02 orthotopic tumor‐bearing mice (Figure 1F). The most striking tumor inhibition effect was observed in the subcutaneous H22 tumors treated with HBO+PD‐1 Ab. Figures S5 and S6 (Supporting Information) illustrate a complete elimination of the tumor (5 of 8 mice) in the HBO+PD‐1 Ab treated group even when the tumor volume exceeded 500 mm^3^. No evident toxicity was detected in all the groups (Figures S11–S14, Supporting Information). The results demonstrated that HBO augments the antitumor effect of PD‐1 Ab in stroma‐rich solid tumors while HBO+PD‐1 Ab was safe to treat cancer patients.

### 2.2 HBO Regulates Mechanical TME in Solid Tumors

**Figure 2:**
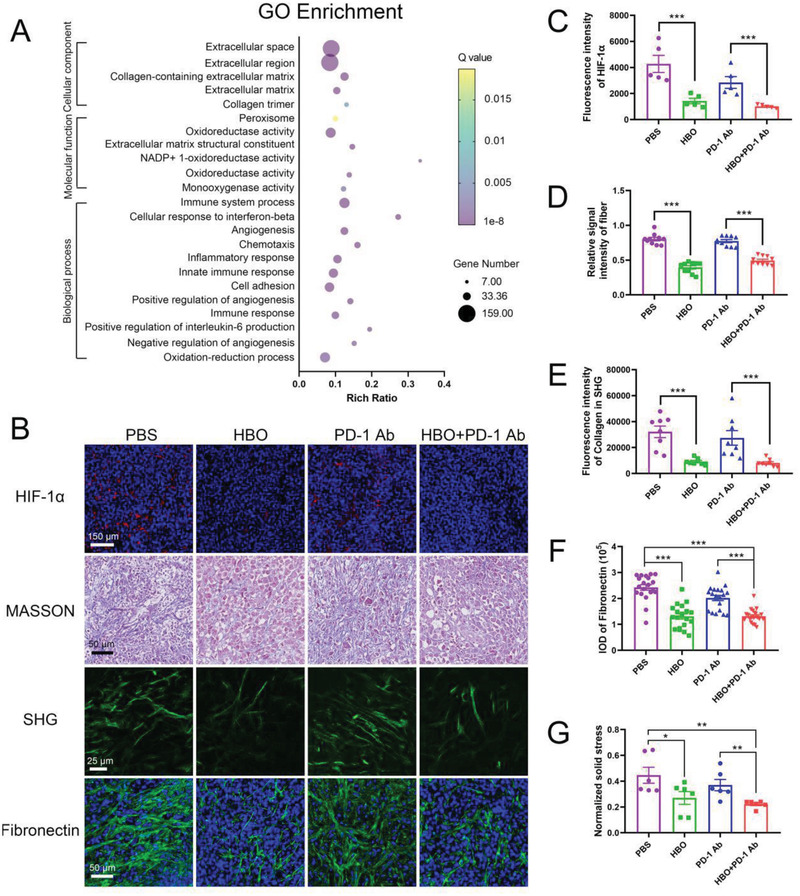
HBO depletes ECM in aberrant mechanical TME. A) GO enrichment analysis of the differentially expressed genes between HBO group and control group. B) Representative immunofluorescence staining of HIF‐1*α* images, MASSON staining images, second harmonic generation (SHG) images, and immunofluorescence staining of fibronectin images of tumor tissue. Quantification of HIF‐1*α* C), collagen in MASSON staining D), and SHG E), fibronectin in immunofluorescence staining F) of tumor tissue sections, respectively. G) Normalized solid stress of 4T1 orthotopic tumors after various treatments. Error bars indicate SEM (*n* = 20). Statistical significance was calculated by *t*‐test. *P*‐values: *, *P* < 0.05; **, *P* < 0.01; ***, *P* < 0.001.

Differentially expressed genes between the HBO‐treated and the untreated control groups were evaluated with transcriptome sequencing of orthotopic H22 tumors to understand how HBO magnifies the antitumor effects of PD‐1 Ab in desmoplastic solid tumors. Figure S15 (Supporting Information) outlines the analysis procedures used in the study. Of the 53269 genes detected, 874 showed significant differential expression between the HBO and control groups. It was also observed that HBO modulated the genes linked to ECM components, oxidation‐reduction, immune responses, and angiogenesis (Figure S16, Supporting Information), demonstrating the HBO influence on TME of orthotopic H22 tumors. The results indicated that HBO might improve antitumor efficacy of PD‐1 Ab via multiple mechanisms. Gene ontology (GO) term enrichment analysis indicated that the most enriched genes involved with cellular components included collagen‐containing ECM, ECM, collagen trimer, and fibrillary collagen trimer (**Figure** **2**A). The expressions of HIF‐1*α* (an indicator of tumor hypoxia), collagen, fibronectin, and elastin (three main components in ECM) were analyzed further to confirm HBO's influence on ECM. Immunofluorescence staining revealed that HIF‐1*α*, collagen fiber, collagen I, and fibronectin decreased significantly with HBO treatment, whereas the amount of elastin remained unchanged (Figure 2B–F; and Figure S17, Supporting Information). Second harmonic generation (SHG) imaging also showed a decreased collagen content (Figure 2B,E). To further uncover the biological consequences of HBO treatment on TME mechanics, solid stress was measured and quantified in orthotopic 4T1 tumors using the tumor opening approach;^[^ ^32^, ^33^ ^]^ the solid stress declined remarkably post‐HBO treatment (Figure 2G; and Figure S18, Supporting Information). Figure 2B–G shows that PD‐1 Ab exerts negligible impact on ECM, whereas the combined HBO+PD‐1 Ab modulates ECM through HBO. Collectively, these findings confirm that HBO modulates aberrant mechanical TME by depleting collagen fiber, collagen I, and fibronectin in ECM, thereby releasing solid stress in solid tumors.

### 2.3 HBO Promotes PD‐1 Ab Delivery and T Cell Infiltration

**Figure 3:**
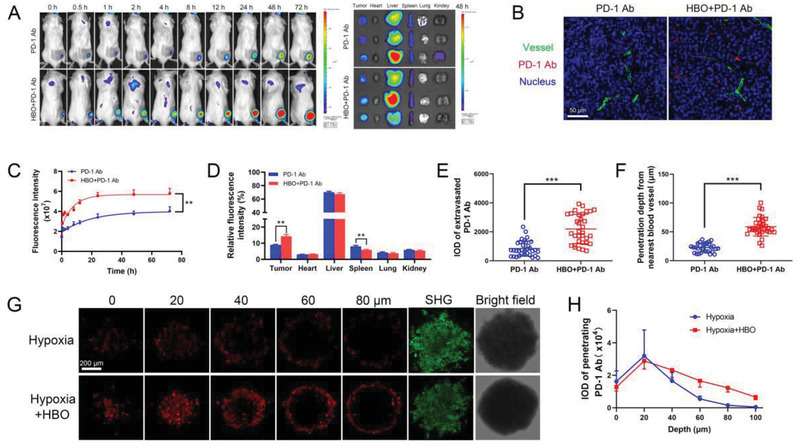
HBO facilitates PD‐1 Ab delivery. A) In vivo fluorescence imaging of mice after intravenous injection of PD‐1 Ab and ex vivo fluorescence imaging of tumors and major organs 48 h post administrations. B) In vivo penetration of PD‐1 Ab in H22 subcutaneous tumor tissue of different treatments. The scale bar is 50 µm. C) Semiquantification of the fluorescence intensity in tumor site at different time points (*n* = 5). D) Organ and tumor accumulation of PD‐1 Ab 48 h postinjection (*n* = 5). E) Average fluorescence intensity of extravasated PD‐1 Ab in tumor tissue (*n* = 20). F) The penetration depth of PD‐1 Ab from the nearest blood vessel (*n* = 20). G) Representative penetrated PD‐1 Ab fluorescence images, SHG, and bright field images of 4T1‐SS. H) Average fluorescence intensity of penetrated PD‐1 Ab in 4T1‐SS (*n* = 3). Error bars indicate SEM. Statistical significance was calculated by *t*‐test. *P*‐values: *, *P* < 0.05; **, *P* < 0.01; ***, *P* < 0.001.

**Figure 4:**
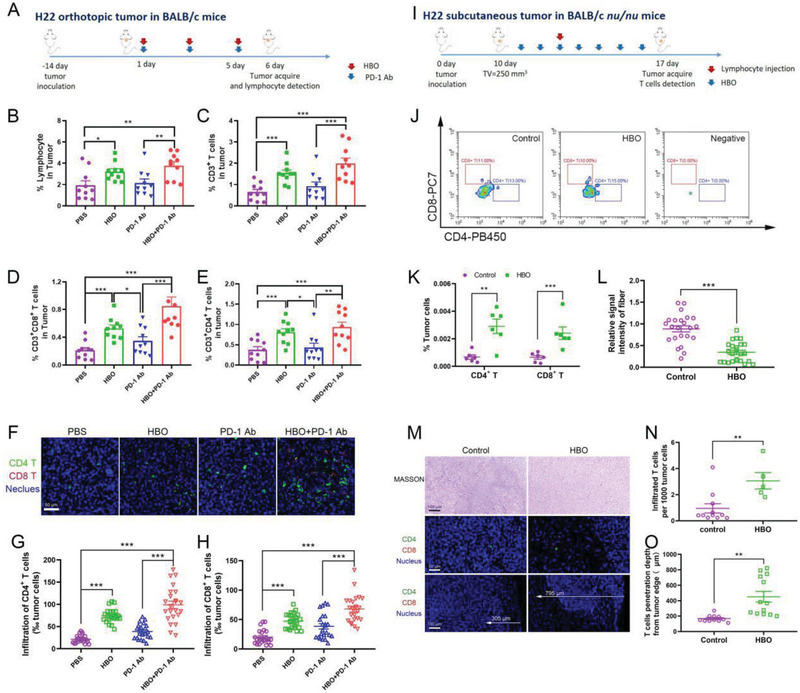
HBO enhances T cell infiltration into tumor parenchyma. A) Experimental scheme of HBO enabling T cell infiltration in H22 orthotopic tumor‐bearing BALB/c mice. Proportion of infiltrated CD45^+^ lymphocyte B), CD3^+^ T cells C), CD8^+^ T cells D), and CD4^+^ T cells E) in tumors (*n* = 10). F) Immunofluorescence staining of CD4^+^ T cell and CD8^+^ T cell images. The scale bar is 50 µm. Quantification of CD4^+^ T cells G) and CD8^+^ T cells H) in tumor tissue sections (*n* = 20). I) Experimental procedures of HBO enhancing T cell infiltration in H22 subcutaneous tumor‐bearing BALB/c *nu/nu* mice. J,K) Infiltrated CD4^+^ T cells and CD8^+^ T cells in tumors were detected by flow cytometry (*n* = 6) after adoptive lymphocytes transfer. M) MASSON staining images, immunofluorescence staining images of T cells (CD4^+^ and CD8^+^) of tumor tissue. L) Quantification of collagen in MASSON staining of tumor tissue sections (*n* = 20). N) Average infiltrated T cells in tumor tissue (*n* = 20). O) The infiltration depth of T cells from the tumor edge (*n* = 20). Error bars indicate SEM. Statistical significance was calculated by *t*‐test. *P*‐values: *, *P* < 0.05; **, *P* < 0.01; ***, *P* < 0.001.

The dense ECM surrounding tumor nests excluded PD‐1 Ab and T cells infiltration due to their size; the diameter of PD‐1 Ab was ≈14 nm (Figure S19, Supporting Information), while T cell had a size of some dozens of micrometers. HBO might facilitate the PD‐1 Ab delivery and T cells infiltration into tumor parenchyma by depleting ECM (Figure 2). To confirm this hypothesis, we examined the influence of HBO on PD‐1 Ab delivery. For observation convenience, we conjugated PD‐1 Ab with the fluorescent dye Dylight680, which emitted fluorescence at 710 nm and was ideal for in vivo imaging of PD‐1 Ab. We intravenously injected the Dylight680 labeled PD‐1 Ab to H22 subcutaneous tumor bearing mice to track the distribution of PD‐1 Ab as a function of time. As displayed in **Figure** **3**A, higher fluorescence can be detected in images with HBO treatment than that without, suggesting HBO is advantageous for PD‐1 Ab tumor accumulation. Semiquantification of Figure 3A is shown in Figure 3C, which consistently supports that more PD‐1 Ab is accumulated in HBO+PD‐1 Ab. The distribution of PD‐1 Ab in tumors and major organs was evaluated with ex vivo imaging, and a higher fluorescence was detected in HBO+PD‐1 Ab 48 h postinjection (Figure 3A,D), reinforcing HBO to be conducive to PD‐1 Ab tumor accumulation. The tumor vessels were stained with CD31 antibodies to characterize extravasation and penetration of Dylight680 labeled PD‐1 Ab from tumor blood vessels (Figure 3B). The quantified fluorescence intensities in more than 10 images showed increased mean fluorescence intensity from 843 for PD‐1 Ab to 2192 for HBO+PD‐1 Ab, suggesting that HBO raised the PD‐1 Ab extravasation by 2.6 times (Figure 3E). The HBO‐treated PD‐1 Ab also showed 2.6 times increased penetration from the nearest blood vessels (Figure 3F). The enhanced extravasation and penetration of PD‐1 Ab was most likely a consequence of HBO‐induced ECM degradation (Figure 2). Furthermore, we established a stroma‐rich spheroid model consisting of 4T1 cancer cells and NIH‐3T3 fibroblasts (4T1‐SS) to reveal the impact of ECM depletion on PD‐1 Ab penetration (Figure 3G). The SHG imaging showed that the HBO‐treated 4T1‐SS reported 40% decrease in collagen, facilitating deeper PD‐1 Ab penetration (Figure 3G,H). **Figure** **4**A shows the experimental outlines for investigating the influence of HBO on the infiltration of lymphocytes, especially T cells, into tumor parenchyma in the study. Flow cytometry analysis revealed that HBO uplifts the infiltrations of lymphocytes (Figure 4B), T cells (Figure 4C), CD8^+^ T cell (Figure 4D), and CD4^+^ T cell (Figure 4E) into the tumor bed of orthotopic H22 tumors. It was also observed that the enhancement was a direct consequence of the HBO treatment; PD‐1 Ab alone had little effect. These conclusions were validated by immunofluorescence staining images too (Figure 4F–H). We established an H22 subcutaneous tumor model in BALB/c nude mice to demonstrate a causality relationship between HBO treatment and T cell infiltration, and observed the infiltration of adoptive transferred lymphocytes. Figure 4I shows the experimental procedure. Without transferred lymphocytes, negligible T cells can be detected in the negative group, justifying the success of this model. Figure 4J,K critically substantiate the conclusion that HBO facilitates the infiltration of T cells, including both CD4+ and CD8+ T cells. HBO also lessened the collagen in nude mice (Figure 4M,L), raised the number of infiltrated T cells from 0.95 to 3.06 per 1000 tumor cells, and increased the mean infiltration distance from 167 to 451 µm (Figure 4M–O). Thus, by depleting ECM in stroma‐rich solid tumors, HBO not only augmented the accumulation and penetration of PD‐1 Ab but also the infiltration of T cells into tumor parenchyma.

### 2.4 HBO Helps PD‐1 Ab Trigger Robust Immune Responses

**Figure 5:**
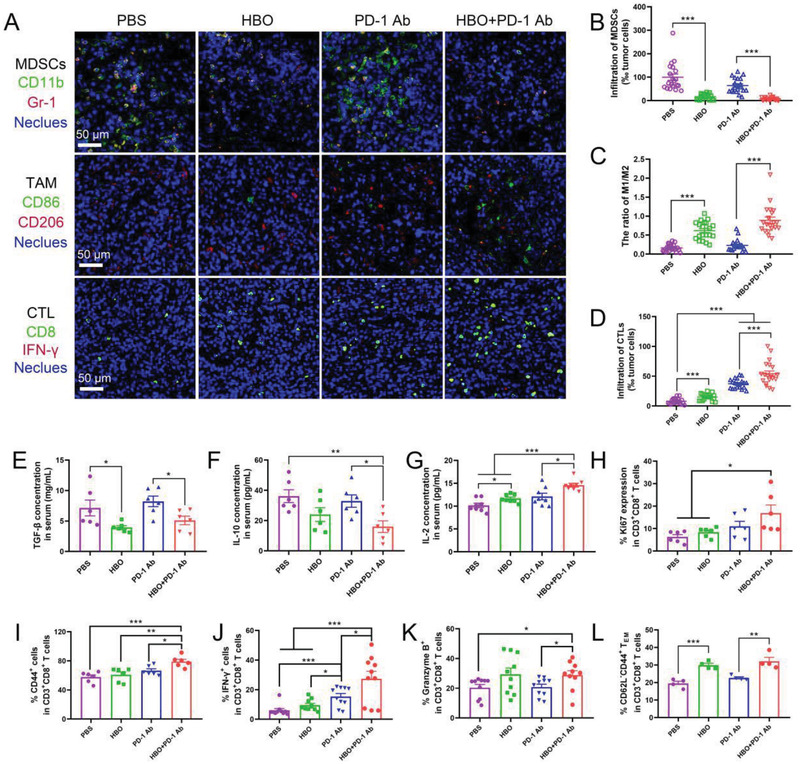
HBO reprograms immune TME and helps PD‐1 Ab invigorate CTLs. A) Immunofluorescence staining of MDSCs (CD11b and Gr‐1 colocalization), TAM (M1: CD86, M2: CD206) images and CTL (CD8 and IFN‐*γ* colocalization) images. The scale bar is 50 µm. Quantification of MDSCs B), ratio of M1/M2 TAMs C), and CTL D) in tumor tissues sections (*n* = 20). Cytokine levels of E) TGF‐*β*, F) IL‐10, and G) IL‐2 in serum from H22 orthotopic tumor‐bearing mice analyzed by ELISA (*n* = 6). Ki67 expression H), CD44 I), IFN‐*γ* J), granzyme B K), and effector memory T cells L) proportions in H22 orthotopic tumor immediately after various treatments analyzed by flow cytometry. Error bars indicate SEM. Statistical significance was calculated by *t*‐test. *P*‐values: *, *P* < 0.05; **, *P* < 0.01; ***, *P* < 0.001.

**Figure 6:**
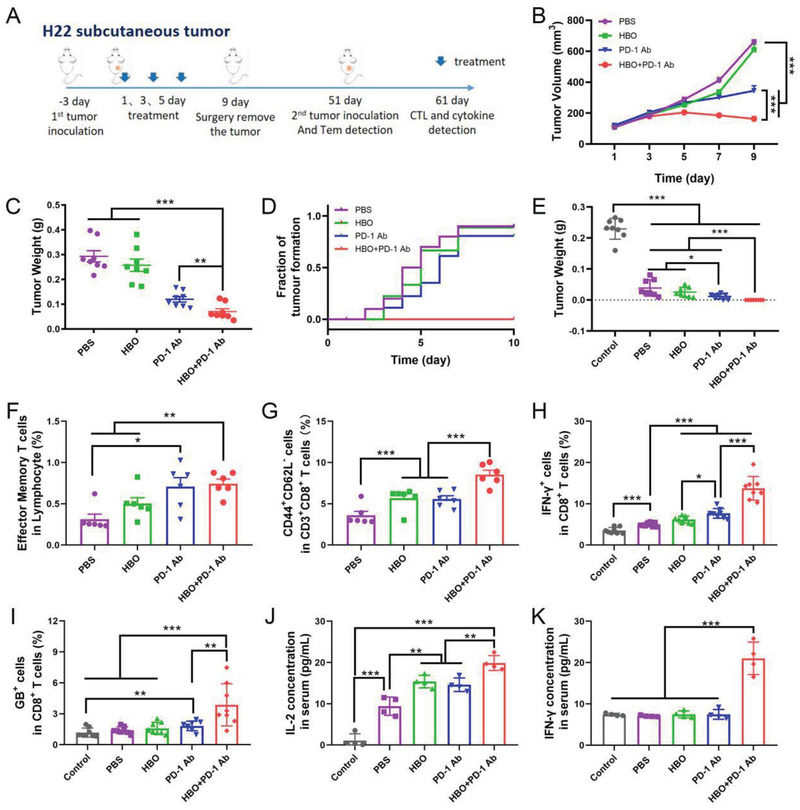
HBO synergizes with PD‐1 Ab to inhibit the relapse of the H22 tumor. A) Experimental scheme of HBO helping PD‐1 Ab trigger a long‐term immunological memory effect. B) Tumor growth curves and D) tumor weight of the first tumors (*n* = 8). C) Tumor formation curves and E) tumor weight of the rechallenged tumors (*n* = 8). Proportions of effector memory T cells gated on lymphocyte F) and gated on CD8^+^ T cells G) from spleen analyzed by flow cytometry at day 51 before mice were rechallenged with the secondary tumors (*n* = 6). IFN‐*γ* H) and granzyme B I) expression of CD8^+^ T cells in lymph node from mice isolated 10 days after mice were rechallenged with the secondary tumors (at day 61) (*n* = 8). Cytokine of J) IL‐2 and K) IFN‐*γ* in serum from mice isolated 10 days after mice were rechallenged with the secondary tumors (at day 61) (*n* = 4). Error bars indicate SEM. Statistical significance was calculated by *t*‐test. *P*‐values: *, *P* < 0.05; **, *P* < 0.01; ***, *P* < 0.001.

HBO facilitated the infiltration of PD‐1 Ab and T cells into tumor parenchyma of stroma‐rich solid tumors (Figures 2, 3, 4). Nonetheless, these were insufficient for potent CTLs and robust immune responses because solid tumors are known for hypoxia‐induced immunosuppression.^[^ ^14^, ^15^ ^]^ Therefore, we evaluated the impact of HBO on immune TME and T cell‐mediated immune responses. HBO relieved tumor hypoxia (Figure 2B,C), thereby decreasing the PD‐L1 expression in H22 orthotopic tumor accordingly (Figure S20, Supporting Information). HBO suppressed the MDSCs infiltrations (**Figure** **5**A,B; and Figure S21, Supporting Information), an observation consistent with an earlier study disrupting tumor hypoxia with TH‐302.^[^ ^19^ ^]^ In this study, HBO increased the ratio between M1 and M2 phenotype macrophage (Figure 5A,C; and Figure S21, Supporting Information) and significantly decreased T_reg_ in tumor tissues (Figure S22, Supporting Information), which agreed well with observations in lung cancers.^[^ ^18^ ^]^ Thus, HBO inhibited suppressive immune cells by overcoming tumor hypoxia. It was also observed that inhibitory cytokines such as IL‐10 and TGF‐*β* that are mainly secreted by MDSCs, M2 TAMs, and T_reg_ were considerably reduced in both serum (Figure 5E,F) and tumor tissues (Figure S23, Supporting Information) after HBO treatment, indicating it is HBO, not PD‐1 Ab, that reprograms TME from being immunosuppressive to immunostimulatory. Furthermore, immunosuppressive signaling molecule cyclic adenosine monophosphate (cAMP) upsurged in PD‐1 Ab group indicating adaptive resistance but diminished post‐HBO treatment (Figure S24, Supporting Information). The study also noted that HBO promoted the infiltration of IFN‐*γ* positive CTLs and the stimulatory cytokines, including IL‐2 and IFN‐*γ* (Figure 5A,D,G). The highest values were obtained in HBO+PD‐1 Ab. We further analyzed the key functions of CTLs by flow cytometry; Figure S25 (Supporting Information) shows the clustering diagram details. It was observed that HBO cooperated with PD‐1 Ab to induce the most potent CTLs in terms of proliferation, activation, and tumor elimination (Figure 5H–K). The results displayed in Figures 4 and 5 collectively indicate that HBO selectively promotes the infiltration and functional maintenance of CTLs but suppresses suppressive immune cells, including MDSCs, M2 TAMs, and T_reg_. As such, CTLs has been unleashed, accounting for the augmented antitumor effects of PD‐1 Ab observed in Figure 1. We also found considerably raised effector memory T (T_em_) cells after HBO treatment (Figure 5L), implying that HBO enabled PD‐1 Ab to suppress tumor relapses. For this reason, we established a recurrence model of H22 subcutaneous tumor to study the long‐term immunological memory effect.^[^ ^34^ ^]^ **Figure** **6**A outlines the experimental scheme. We inoculated the first tumors and treated the mice. Consistent with Figure 1; and Figures S5 and S6 (Supporting Information), HBO potentiated the curative effect of PD‐1 Ab (Figure 6B,D). We removed the first tumors surgically, waited for 6 weeks to ensure that the CTLs generated by the first tumors were no longer present, and rechallenged the mice with the second tumors to imitate the recurrence of clinical tumors. No tumor forms in HBO+PD‐1 Ab (Figure 6C,E; and Figures S26, Supporting Information). In striking contrast, tumors were formed in the other three groups, PBS, HBO, and PD‐1 Ab, though the second tumors in these groups were smaller than those without inoculating the first tumors. These results suggest that HBO synergizes with PD‐1 Ab to inhibit tumor relapses. To further verify if the impaired tumorigenicity was caused by the immunological memory effect,^[^ ^34^ ^]^ the proportion of T_em_ in mice spleen was characterized before inoculating the second tumors (at day 51). We found that HBO+PD‐1 Ab had the highest proportion of T_em_ in lymphocytes (Figure 6F) and T cells (Figure 6G). Besides, flow cytometry analysis of lymph node 10 days after inoculating the second tumors also revealed that HBO+PD‐1 Ab exhibited the highest proportion of CTLs in secreting IFN‐*γ* (Figure 6H; and Figure S27, Supporting Information) and granzyme B (Figure 6I; and Figure S27, Supporting Information). The highest levels of stimulatory cytokines, IL‐2 and IFN‐*γ*, were also detected in the serum of HBO+PD‐1 Ab (Figure 6J,K). Collectively, these results illustrated that HBO reprograms the immune TME and synergizes with PD‐1 Ab to trigger robust CTLs for tumor cell eradication and long‐term immunological memory effect against tumor relapses.

### 2.5 HBO Boosts PD‐1 Ab Antitumor Effect Toward Clinical Samples

**Figure 7:**
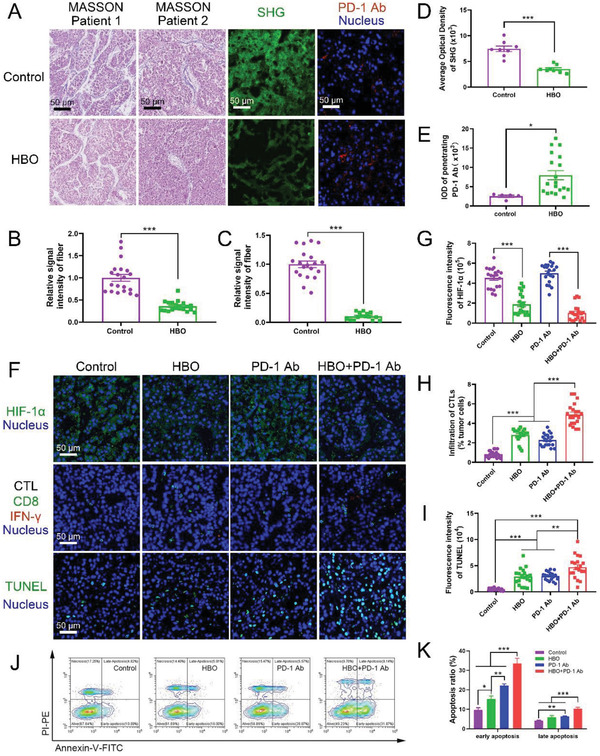
HBO depletes ECM and boosts antitumor effect of PD‐1 Ab toward clinical samples. MASSON staining images, SHG images, and the penetrated PD‐1 Ab in clinical tumor samples A). The scale bar is 50 µm. Quantification of collagen fiber of two patients in MASSON staining B,C), SHG D), and penetrated PD‐1 Ab E) in clinical tumor sample sections, respectively (*n* = 20). F) Representative immunofluorescence staining of HIF‐1*α* images, CTL staining images, and TUNEL staining images in clinical tumor samples. The scale bar is 50 µm. Quantification of HIF‐1*α* G), CTL H), and apoptosis index I) in clinical tumor sample sections, respectively (*n* = 20). J,K) The antitumor effect of HBO+PD‐1 Ab on clinical tumor samples was evaluated by cell apoptosis through Annexin V and PI staining (*n* = 8). Error bars indicate SEM. Statistical significance was calculated by *t*‐test. *P*‐values: *, *P* < 0.05; **, *P* < 0.01; ***, *P* < 0.001.

The study also explored if HBO promoted antitumor effect of PD‐1 Ab toward clinical samples. To that end, we tested HBO and PD‐1 Ab in tumor tissues surgically removed from HCC patients (Figure S29 and Table S1, Supporting Information). Consistent with the results obtained from the rodent tumor tissues, the expression of HIF‐1*α* in clinical samples decreased substantially after treatments with HBO three times (**Figure** **7**F,G), the collagen content decreased too (Figure 7A–D). Thus, HBO promoted the infiltrations of both PD‐1 Ab (Figure 7E) and CTLs (Figure 7F,H), derived from the peripheral blood mononuclear cells of HCC patients, into clinical tumor tissues, contributing to the highest apoptosis ratio for HBO+PD‐1 Ab (Figure 7F,I). As seen in Figure 7J,K, HBO helped PD‐1 Ab achieve the highest ratios in both early‐ and late apoptosis of the HCC patient's cancer cells. To summarize, HBO depleted ECM, facilitated PD‐1 Ab penetration, and promoted tumor cell eradication of CTLs against tumor cells derived from HCC patients (Figure 7).

## 3 Discussion

For the first time, we have used HBO to explore PD‐1 Ab‐mediated immune responses against various solid malignancies, such as HCC, PDAC, and TNBC that share common aberrant mechanical properties and hypoxia‐induced immunosuppression. The study reveals the mechanistic understanding of HBO's dual benefits to PD‐1 Ab. First, HBO significantly depleted ECM's major components, thereby facilitating the delivery of PD‐1 Ab and the infiltration of CTLs into tumor parenchyma. Second, by overcoming tumor hypoxia, HBO reprogrammed TME from being immunosuppressive to immunopermissive and empowered PD‐1 Ab to inhibit the relapses of second tumors. The two benefits complement each other. HBO synergistically potentiated the antitumor effects of PD‐1 Ab against a wide spectrum of rodent stroma‐rich solid tumors and cancer cells derived from HCC patients.

It is important to note that HBO is capable of regulating the aberrant mechanical TME. However, HBO might deplete excessive ECM via multiple mechanisms. Previously, we identified that HBO decreased collagen deposition via the hypoxia/HIF‐1*α*/CTGF/collagen I signaling axis.^[^ ^30^ ^]^ In another instance, we confirmed that HBO remodeled ECM by generating excessive ROS.^[^ ^31^ ^]^ The idea that ROS could degrade ECM has been substantiated by numerous recent studies.^[^ ^35^ ^]^ In this study, the results (Figure S16, Supporting Information) demonstrated that the genes related to oxidation–reduction were significantly changed post‐HBO treatment, confirming that ROS played a significant role during HBO therapy.^[^ ^36^ ^]^ Figure S16 (Supporting Information) also highlighted matrix metalloproteinase‐7 (MMP‐7) gene had increased after HBO. Crucially, Figure S30 (Supporting Information) confirmed that the expressions of MMP‐7 were upregulated in tumor tissues from both rodent HCC H22 tumor and clinical tumor tissues derived from HCC patients. Nonetheless, the producers of MMP‐7 are unclear and remain to be studied in detail in the future while the biological implications of upregulated MMP‐7 merit additional investigations.^[^ ^37^ ^]^ Figure S16 (Supporting Information) further revealed that HBO therapy reduced the genes related to the CXCL12/CXCR4 signaling axis, an observation in agreement with recent studies that reported blocking CXCR4 decreased collagen content and promoted CTLs infiltration.^[^ ^24^, ^38^ ^]^ Besides, TGF‐*β* signaling pathway is critically involved in the synthesis of collagen,^[^ ^39^ ^]^ the reduced gene (Figure S16, Supporting Information) and expression (Figure 5E) of TGF‐*β* may have contributed to the diminished ECM. Although the operative mechanism is unclear yet, it could be safely suggested that HBO depleted ECM in tumor tissues for both rodent HCC H22 tumor tissues (Figure 2) and clinical samples derived from HCC patients (Figure 7), paving the road for the PD‐1 Ab and CTLs infiltrations (Figures 3, 4, 5, and 7).

Our results confirmed the notion that disrupting tumor hypoxia is beneficial for ICB mediated immunotherapy,^[^ ^18^, ^19^, ^23^, ^24^, ^27^ ^]^ as exemplified with reduced MDSCs, M2 TAMs, T_reg_, TGF‐*β*, and IL‐10 and strengthened CTLs (Figure 5) and T_em_ (Figures 5 and 6). HBO also extended the ICB applications to stroma‐rich solid tumors (Figures 1, 2, 3, 4, and 7). The HBO therapy possessed two unique merits over other hypoxia disruption strategies. The oxygen molecules used in overcoming hypoxia are much smaller than metformin, TH‐302, and the diverse oxygen‐carrying nanodrug delivery systems (NDDS). They possess a higher capacity to penetrate the dense ECM surrounding the tumor nests to mitigate hypoxia distant from the blood vessels. To this end, HBO and respiratory hyperoxia would be the two potential choices.^[^ ^21^, ^29^ ^]^ Compared with respiratory hyperoxia, for instance breathing 60% oxygen, HBO operates at a pressure higher than normal atmosphere, usually at 2.5 ATA or 3 ATA, at which circumstance the delivery of oxygen is not limited by hemoglobin in blood.^[^ ^40^ ^]^ Thus, HBO might be more effective than respiratory hyperoxia in enhancing oxygen diffusion in chaotic blood vessels of solid tumors.^[^ ^21^, ^29^ ^]^ Nonetheless, a strict and complete comparison between HBO and other hypoxia disruption strategies is warranted. From a clinical translation viewpoint, HBO is a good choice among various hypoxia disruption strategies. Though metformin has been used as a diabetic drug for many years, its influence on antitumor immunity is multifactorial.^[^ ^41^ ^]^ While most oxygen‐carrying NDDS is far from being applied in clinical settings and TH‐302 needs to undergo clinical tests, HBO has been applied in the clinic for over 100 years, with 13 types of indications approved by the Underwater and Hyperbaric Medical Society besides the well‐documented side effects and contradictions.^[^ ^40^ ^]^ The use of HBO in combination with radiotherapy and photodynamic therapy to combat hypoxic tumors has been tested. Although the antitumor efficacy of these combinations is modest, the application of HBO to cancer patients appears to be safe.^[^ ^28^ ^]^

## 4 Conclusion

We envision HBO together with PD‐1 Ab might provide additional benefits to patients suffering from various solid tumors. However, translating the findings of this study to bedside applications requires optimization of the sequencing and dosing of HBO+PD‐1 Ab. Notwithstanding these difficulties and hurdles, the combination of HBO and PD‐1 Ab merits clinical trial to test its safety and efficacy for cancer patients of stroma‐rich hypoxic solid tumors. In this study, we demonstrated that HBO promotes antitumor effect of PD‐1 Ab toward HCC, PDAC, and TNBC. We propose that other types of solid tumors featured with dense ECM and severe hypoxia, for instance, glioblastoma and prostate cancer, might also benefit from this new combination therapy. Given that HBO remodels TME and enables T cells infiltration into tumor bed by depleting dense ECM, we also expect HBO to be beneficial for adoptive cell therapy (ACT) such as endogenous T‐ and NK cells, genetically engineered T cell, NK cell, and macrophage, in terms of their infiltration and function maintenance in the hostile TME of stroma‐rich solid malignancies.^[^ ^42^, ^43^, ^44^ ^]^ Studies on the combination of HBO and ACT are currently under progress and will be reported in the future.

## 5 Experimental Section

### Animals

BALB/c mice, BALB/c *nu/nu* mice, and C57 mice were purchased from Beijing Vital River Laboratory Animal Technology Co. Ltd., Beijing, China. These animals were housed in a specific pathogen‐free environment. All animal experiments were performed in accordance with the internationally accepted principles and Guidelines for the Care and Use of Laboratory Animals of Huazhong University of Science and Technology. The experiment protocols were approved by the Institutional Animal Ethical Committee of the Huazhong University of Science and Technology. The animal studies performed were IACUC approved.

### Cell Lines and Reagents

4T1, H22, HepG2, NIH‐3T3, and Panc02 were purchased from BeNa Culture Collection, China. Anti‐mouse PD‐1 antibody (PD‐1 Ab, BE0146‐100 mg) was purchased from Bio X Cell. Anti‐human PD‐1 Ab was obtained as a gift from Livzon Pharmaceutical Group Inc., China. Flow cytometry antibodies included PE anti‐mouse CD274 (B7‐H1, PD‐L1) (124308), APC/Cyanine7 anti‐mouse CD45 (103132), FITC anti‐mouse CD3 (100204), Brilliant Violet 421 anti‐mouse CD4 (100563), PE/Cy7 anti‐mouse CD8a (100722), PE anti‐mouse IFN‐*γ* (505808), Alexa Fluor 647 anti‐human/mouse Granzyme B (515406), APC anti‐mouse Ki‐67 (652406), PE anti‐mouse/human CD44 (103008), PE anti‐mouse Ly‐6G/Ly‐6C (Gr‐1) (108407), APC/Cyanine7 anti‐mouse/human CD11b (101226), Brilliant Violet 421 anti‐mouse F4/80 (123137), PE anti‐mouse CD86 (105008), PE/Cy7 anti‐mouse CD206 (MMR) (141720), Alexa Fluor 647 anti‐mouse FOXP3 (126408), APC anti‐mouse CD49b (pan‐NK cells) (108910), PE anti‐mouse CD25 (102008), PE anti‐mouse/human CD44 (103008), APC anti‐mouse CD62L (104412), Zombie Aqua Fixable Viability Kit (423102), Cell Activation Cocktail (with Brefeldin A) (423304), True‐Nuclear Transcription Factor Buffer Set (424401), and Intracellular Staining Permeabilization Wash Buffer (10X) (421002) were purchased from BioLegend.

### Fluorescent Labeling of PD‐1 Ab

Cy5‐conjugated PD‐1 Ab was prepared as follows. PD‐1 Ab was conjugated with CY5‐NHS ester (Xi'an RuiXin Biological: R‐FR‐005) at a molar ratio of 1:1 and the reaction mixture was dialyzed in PBS for 24 h to remove the unconjugated CY5‐NHS. Dylight680 labeled PD‐1 Ab was prepared by using a Lightning‐Link Rapid DyLight 680 Labeling Kit (Expedeon: 327‐0015). First, 1 µL of LL‐Rapid modifier reagent was added to 10 µL PD‐1 Ab and mixed gently. The mixture was pipetted directly into the lyophilized LL‐Rapid mix. After incubating for 15 min, 1 µL of LL‐Rapid quencher reagent was added to the reaction solution. The dylight680 conjugated PD‐1 Ab thus obtained was ready for use after 5 min of the reaction.

### H22 Subcutaneous Tumor Model

Mice were injected with single‐cell suspension of 1 × 10^6^ H22 cells per mouse subcutaneously (S.C.) on the right flank as described previously.^[^ ^30^ ^]^ When tumor volume increased to 100 mm^3^, 32 mice were divided into four groups, 8 mice per group, and the day was recorded as day 1. The groups were as follows: PBS, HBO, PD‐1 Ab, and HBO+PD‐1 Ab. Mice in the PBS group and the PD‐1 Ab group were injected with 100 µL PBS or 100 µL (1 mg mL^−1^) PD‐1 Ab intravenously (I.V.) by tail vein on day 1, 3, and 5. Mice in the HBO group and HBO+PD‐1 Ab group received the HBO treatment once on day 1, 3, and 5. HBO treatment was performed through a small animal hyperbaric chamber, which was pumped with pure oxygen to a pressure of 2.5 atm. After 1.5 h, the chamber was slowly deflated to return to normal atmospheric pressure. Mice in the HBO+PD‐1 Ab group were injected with PD‐1 Ab (100 µL, 1 mg mL^−1^) by tail vein 2 h after receiving the HBO treatment. After removal from the HBO chambers, the mice were allowed to breathe in the room air. Tumor size was measured using a caliper to determine the diameter: longest surface length (*L*), width (*W*), and the tumor size was expressed as volume (*L*x*W* ^2^/2). The tumor size was monitored every 2 days. The mice were then sacrificed at day 18 and the tumors were removed, photographed, and weighed. The antitumor efficiency was also evaluated by histological examination. People who assisted with the measurements, histological studies, and other analyses were blinded to experimental group. The tumors were taken out from the mice and fixed in 4% paraformaldehyde solution. The fixed tumors were embedded with paraffin and sectioned into thin pieces, and then stained with Hematoxylin and Eosin staining (H&E staining), Ki67 staining (Biossci: PA1007), and TUNEL (terminal deoxynucleotidyl transferase dUTP nick end labeling, Rohce: 11684817910). The histopathological alterations of tumor tissues and cells were characterized under a light microscope and a confocal laser scanning microscope (Olympus, CX‐21). Major organs including heart, liver, spleen, lung, and kidney in each group were also collected and examined by H&E staining.

### H22 Orthotopic Tumor Model

H22 subcutaneous tumor tissue was acquired and cut into pieces (2–4 mm) in a sterile dish. The tumor pieces were then implanted into the liver of healthy mice by surgery. All mice were randomized into four groups, 2 weeks postsurgery, and then they were treated, respectively. The treatment protocol was the same as that of the H22 subcutaneous tumor model.

### Panc02 Orthotopic Tumor Model

The pancreas of mice was exposed by surgery, and then injected with single‐cell suspension of 1 × 10^6^ Panc02 cells per mouse into the cauda of pancreas. All mice were randomized into four groups, 1 week postsurgery, and then they were treated, respectively. The treatment protocol was the same as that of the H22 subcutaneous tumor model.

### 4T1 Orthotopic Tumor Models

The skin around the right fourth abdomen of mice was cut by surgery, and then injected with single‐cell suspension of 1 × 10^6^ 4T1 cells per mouse on the abdomen adipose pad. The grouping and treatment protocol was the same as that of the H22 subcutaneous tumor model.

### Immunofluorescence Staining and Imaging

Mice tumor tissues were fixed and embedded in paraffin, and sections with 10 µm thickness were prepared. For characterization of tumor hypoxia, the tumor sections were immunofluorescence stained with anti‐HIF‐1*α* FITC‐conjugated antibody (PTG: 20960‐1‐AP) and imaged under a confocal fluorescence microscope. For characterization of ECM, MASSON staining, Sirius red staining, collagen I (Biossci: PA1026) immunofluorescence staining, and second harmonic generation (SHG) imaging were used to detect collagen in ECM. Fibronectin (abcam: ab199056) and elastin fibers in ECM were also probed with immunofluorescence staining and Verhoeffe‐Van Gieson (VVG) staining. To evaluate the infiltration of immune cells into tumor parenchyma, CD4 (Biossci: PA1052), CD8 (Biossci: PA1050), IFN‐*γ*(Bioss: bs‐0480R), CD11b (Biossci: PA1045), Gr‐1 (abcam: ab238132), CD86 (CST: 19589), CD206 (Biossci: PA1052) were introduced to identify CD4^+^ T cells, CD8^+^ T cells, CTLs (CD8^+^IFN‐*γ* ^+^), MDSCs (CD11b^+^Gr‐1^+^), M1 (CD86^+^), and M2 (CD206^+^) tumor associated macrophages by immunofluorescence staining. For quantitation of hypoxia, ECM components, and lymphocytes in tumor tissues, image pro plus software was used to analyze fluorescence signals from the staining images. At least six fields per tumor slide were counted and at least three tumor tissues per group were analyzed.

### Growth‐Induced Solid Stress Measurements

4T1 orthotopic tumor model was established and treated with predetermined protocols. When the tumor diameters reached around 1 cm, the mice were anesthetized by injecting 0.15 mL 1% sodium pentobarbital solution intraperitoneally. Subsequently, each tumor was excised and washed with PBS, and the 3D and weight were measured. To measure solid stress induced opening, the tumor was cut along its longest axis (≈80% of its thickness). The tumor was then allowed to relax for 10 min to reduce any transient poroelastic response. The opening was measured in the center and close to the two edges of the cut at the surface of the tumor. Solid stress index was calculated as the ratio of the measured opening to the longest axis. The detailed procedures and principles have been accounted by others.^[^ ^32^, ^33^ ^]^

### PD‐1 Ab Penetration Studies in 3D Stroma‐Rich Spheroids

3D stroma‐rich spheroids containing 4T1 and NIH‐3T3 (4T1‐SS) were generated by an improved hanging drop method.^[^ ^45^ ^]^ Briefly, 4T1 cells were collected and mixed with NIH‐3T3 cells at a ratio of 2:1 and resuspend in a 1640 medium containing 25% methylcellulose (Sigma‐Aldrich: M0512). A small amount (20 µL) of hanging droplet containing 20 000 cells were then incubated for 3 days to form stroma‐rich spheroids. The stroma‐rich spheroids were harvested for SHG analysis and penetration studies. The stroma‐rich spheroids were incubated at 37 °C, 1% O_2_, and 5% CO_2_ for 24 h, and then treated with one‐time HBO. For control group, no HBO treatment was applied, while other protocols remained the same. The ECM of the harvested stroma‐rich spheroids was then detected by SHG. To evaluate the penetration depth of PD‐1 Ab, the HBO treated or untreated stroma‐rich spheroids were exposed to Cy5 conjugated PD‐1 Ab (10 µg mL^−1^) for 6 h and then were characterized by confocal microscopy.

### In Vivo and Ex Vivo Biodistribution of PD‐1 Ab

To track the distribution of PD‐1 Ab in mice, tumor‐bearing mice (*n* = 5) were first treated with HBO and then I.V. injected with dylight‐680 conjugated PD‐1 Ab. The in vivo fluorescence imaging of PD‐1 Ab was obtained by a Xenogen IVIS in vivo imaging system at different time points. To determine the accumulation and localization of PD‐1 Ab in tumors and various organs, mice were sacrificed 48 h postinjection, while the tumors, hearts, livers, spleens, lungs, and kidneys were harvested for ex vivo imaging using a Xenogen IVIS imaging system. The tumors were then embedded in paraffin, and 10 µm thickness tumor sections were prepared. Tumor vessels were identified by staining with the endothelial cell‐specific marker CD31 (Biossci: PA1032), while the tumor sections were characterized using a Zeiss LSM 710 confocal microscope.

### Flow Cytometry Analysis

Six days after the establishment of H22 orthotopic tumor model, the mice were sacrificed and the tumors were harvested to detect lymphocytes in tumor tissues. The tumors were cut as small as possible and supplemented with collagenase IV (Biosharp) and DNase I (Biosharp), and then incubated at 37 °C, 5% CO_2_ for 45 min. The samples were filtered through a 70 µm cell strainer to harvest single‐cell suspensions. The cell samples were then centrifuged, resuspended in 300 µL PBS, and stained with the corresponding antibodies from BioLegend. Flow cytometry data were collected on a 3‐laser, 12‐color BD CyotoFlex cytometer, and analyzed by software of the instrument. For instance, the cells were stained with APC/Cyanine7 anti‐mouse CD45, FITC anti‐mouse CD3, Brilliant Violet 421 anti‐mouse CD4, and PE/Cy7 anti‐mouse CD8a to differentiate CD4^+^ T cells and CD8^+^ T cells. Notably, CD8^+^ T cells could be classified into CTL and T_em_ by further introducing PE anti‐mouse IFN‐*γ* (505808), Alexa Fluor 647 anti‐human/mouse Granzyme B, or PE anti‐mouse/human CD44, APC anti‐mouse CD62L. APC anti‐mouse Ki‐67 and PE anti‐mouse/human CD44 were introduced to identify proliferation and activation of CD8^+^ T cells. APC/Cyanine7 anti‐mouse/human CD11b, Brilliant Violet 421 anti‐mouse F4/80, PE anti‐mouse CD86, and PE/Cy7 anti‐mouse CD206 (MMR) were introduced to identify M1 and M2 phenotypes TAMs. APC/Cyanine7 anti‐mouse/human CD11b and PE anti‐mouse Ly‐6G/Ly‐6C (Gr‐1) were introduced to identify MDSCs. Alexa Fluor 647 anti‐mouse FOXP3 and PE anti‐mouse CD25 were introduced to identify T_reg_ based on CD4^+^ T cells. PE anti‐mouse CD274 (B7‐H1, PD‐L1) was used to detect the PD‐L1 expression on tumor cells.

### Cytokine and Enzyme Measurements

Tumor tissues were homogenized, centrifuged, and the supernatant was collected for analysis. The serum samples were isolated from mice and diluted for analysis. IL‐2 (BioLegend: 1210202), IFN‐*γ* (BioLegend: 1210002), TGF‐*β* (BioLegend: 1217102), IL‐10 (BioLegend: 1211003), cAMP (eBioscience), Arg1 (Reddot Biotech, Catalog No: RD‐Arg‐Mu), iNOS (Nanjing Jiancheng: A014‐1‐2), and MMP‐7 (eBioscience: E‐EL‐M0783c for mouse, E‐EL‐H1449c for human) were analyzed with the corresponding enzyme‐linked immunosorbent assay (ELISA) kits according to the vendors’ protocols.

### Adoptive Transfer of Lymphocytes

Four‐week‐old BALB/c *nu/nu* mice were subcutaneously injected with single‐cell suspension of 1 × 10^6^ H22 cells per mouse on the right flank as described previously. When the tumor volume increased to 250 mm^3^, the mice were divided into three groups, and the day was recorded as day 10. The groups were as follows: control, HBO, and negative. Mice in the HBO group received HBO treatment every day on days 10–17 (2.5 ATA, 1.5 h). Lymphocytes were isolated from the spleen of healthy BALB/c mice. The spleens of mice were cut as small as possible and crushed with a rubber plug. The samples were then filtered through a 70 µm cell strainer to harvest single‐cell suspensions. The lymphocytes were extracted by a mouse lymphocyte extraction kit (Cedarlane: CL5010). The lymphocytes (1 × 10^9^ cells) were then injected intravenously into the mice of control group and HBO group at day 13. No lymphocyte was injected for the negative group. The mice were then sacrificed at day 18 and their tumors were removed. T cells in tumor tissues were detected by flow cytometry and immunofluorescence staining. MASSON staining was also used to detect collagen in ECM.

### Immunological Memory Effect

To study the immunological memory effect after the HBO and PD‐1 Ab combination treatment, H22 subcutaneous tumors were established and treated with the same protocol as the H22 subcutaneous tumor model. The first tumors were removed by surgery when the tumor volume of each group exhibited significant differences. Each group of mice were divided into two parts after 42 days. One part of the mice was sacrificed, and the effector memory T cells (T_EM_:CD3^+^ CD8^+^ CD44^+^ CD62L^−^) in the spleen were analyzed. Another part of the mice was rechallenged with the second batch of H22 cells (5 × 10^6^ cancer cells) on the same day. The growth of the rechallenged tumors was monitored every two days. On day 61 (10 days after inoculating the secondary tumors), lymph nodes were collected from the mice and homogenized into single‐cell suspensions. CTLs in lymph nodes were detected by flow cytometry. ELISA was used to measure the cytokines in serum of the mice receiving different treatments.

### Clinical Tumor Samples

Tumor tissues of hepatocellular carcinoma were acquired from patients in Tongji Hospital after obtaining the informed consent, and were used under good clinical practice approved by the National Medical Products Administration (NMPA). This study was approved by the Clinical Trial Ethics Committee of Huazhong University of Science and Technology. Fresh tumor tissue (after surgical resection) was kept in a growth medium and cut into pieces (≈4–6 mm) in a sterile plate. All tumor pieces were randomized into four groups, and the time point was recorded as 0 h. The groups were as follows: PBS, HBO, PD‐1 Ab, HBO+PD‐1 Ab. The PBS and PD‐1 Ab groups were incubated at hypoxia conditions (37 °C, 1% O_2_, 5%CO_2_). The HBO and HBO+PD‐1 Ab groups received one‐time HBO treatment (2.5 ATA, 1.5 h) immediately and were then transferred to a hypoxia incubator. At a time point of 4 h, the medium of PD‐1 Ab and HBO+PD‐1 Ab groups were added with human PD‐1 Ab (Livzon) until the final concentration was 15 µg mL^−1^. At the time points of 12 and 24 h, the HBO and HBO+PD‐1 Ab groups received another two HBO treatments. The tumor samples were then collected at 26 h.

### Immunofluorescence Staining and Imaging of Clinical Tumor Samples

The collected clinical tumor samples were fixed, embedded in paraffin, and then sectioned into 10 µm thick tumor sections. The tumor sections were then subjected to immunofluorescence staining with anti‐HIF‐1*α* FITC‐conjugated antibody and characterized under a confocal microscope to detect tumor hypoxia. The MASSON staining and SHG imaging were used to characterize ECM. To evaluate the CTLs in clinical tumor samples after different treatments, CD8 and IFN‐*γ* colocalization were evaluated by immunofluorescence staining. For quantitation of hypoxia, ECM, and CTLs, image pro plus software was used to analyze the fluorescence signals from the staining images. At least 10 fields per tumor were counted and at least 3 tumor tissues per condition were analyzed.

### Antitumor Efficacy of Combination Therapy Toward Clinical Tumor Samples

The collected clinical tumor samples were cut as small as possible, supplemented with collagenase IV (Biosharp: BS035A) and DNase I (Biosharp: BS199A), and incubated at 37 °C, 5% CO_2_ for 45 min. The samples were then filtered through a 70 µm cell strainer to harvest single‐cell suspensions. After that, the cells were labeled with annexin V and propidium iodide (PI) by Annexin V‐FITC Apoptosis Detection Kit (Yeasen: 40302ES60), and analyzed by flow cytometry. The clinical tumor samples were taken out after treatments and fixed in 4% paraformaldehyde solution. The fixed tumors were embedded with paraffin and sectioned into thin pieces, and then stained with TUNEL. For quantitation of apoptosis index of tumor samples, image pro plus software was used to analyze fluorescence signals from the staining images. At least ten fields per tumor slide were counted and at least three tumor tissues per group were analyzed.

### Statistical Analysis

Data were presented as mean ± SEM. Statistical significance was calculated by *t*‐test. *P*‐values of <0.05 were considered statistically significant. Statistical analysis was carried out using GraphPad Prism software.

## Conflict of Interest

Z.F.L., X.L.Y., and X.L. have applied for patents related to this study.

## Supporting information

Supporting Information

## Data Availability

The data that support the findings of this study are available from the corresponding author upon reasonable request.

## References

[1] A. Ribas , J. D. Wolchok , Science 2018, 359, 1350. 29567705 10.1126/science.aar4060 PMC7391259

[2] A. Kalbasi , A. Ribas , Nat. Rev. Immunol. 2020, 20, 25. 31570880 10.1038/s41577-019-0218-4 PMC8499690

[3] D. S. Chen , I. Mellman , Immunity 2013, 39, 1. 23890059 10.1016/j.immuni.2013.07.012

[4] H. Mohammadi , E. Sahai , Nat. Cell Biol. 2018, 20, 766. 29950570 10.1038/s41556-018-0131-2

[5] M. Ringelhan , D. Pfister , T. O'Connor , E. Pikarsky , M. Heikenwalder , Nat. Immunol. 2018, 19, 222. 29379119 10.1038/s41590-018-0044-z

[6] W. J. Ho , E. M. Jaffee , L. Zheng , Nat. Rev. Clin. Oncol. 2020, 17, 527. 32398706 10.1038/s41571-020-0363-5 PMC7442729

[7] I. X. Chen , V. P. Chauhan , J. Posada , M. R. Ng , M. W. Wu , P. Adstamongkonkul , P. Huang , N. Lindeman , R. Langer , R. K. Jain , Proc. Natl. Acad. Sci. USA 2019, 116, 4558. 30700545 10.1073/pnas.1815515116 PMC6410779

[8] A. I. Minchinton , I. F. Tannock , Nat. Rev. Cancer 2006, 6, 583. 16862189 10.1038/nrc1893

[9] D. Wang , T. Wang , H. Yu , B. Feng , L. Zhou , F. Zhou , B. Hou , H. Zhang , M. Luo , Y. Li , Sci. Immunol. 2019, 4, eaau6584. 31300478 10.1126/sciimmunol.aau6584

[10] I. Melero , A. Rouzaut , G. T. Motz , G. Coukos , Cancer Discovery 2014, 4, 522. 24795012 10.1158/2159-8290.CD-13-0985 PMC4142435

[11] T. Stylianopoulos , L. L. Munn , R. K. Jain , Trends Cancer 2018, 4, 292. 29606314 10.1016/j.trecan.2018.02.005 PMC5930008

[12] Y. Huang , Y. Chen , S. Zhou , L. Chen , J. Wang , Y. Pei , M. Xu , J. Feng , T. Jiang , K. Liang , S. Liu , Q. Song , G. Jiang , X. Gu , Q. Zhang , X. Gao , J. Chen , Nat. Commun. 2020, 11, 622. 32001695 10.1038/s41467-020-14425-7 PMC6992734

[13] F. Mpekris , M. Panagi , C. Voutouri , J. D. Martin , R. Samuel , S. Takahashi , N. Gotohda , T. Suzuki , P. Papageorgis , P. Demetriou , C. Pierides , L. Koumas , P. Costeas , M. Kojima , G. Ishii , A. Constantinidou , K. Kataoka , H. Cabral , T. Stylianopoulos , Adv. Sci. 2020, 8, 2001917. 10.1002/advs.202001917 PMC7856901 33552852

[14] R. K. Jain , Cancer Cell 2014, 26, 605. 25517747 10.1016/j.ccell.2014.10.006 PMC4269830

[15] E. B. Rankin , J.‐M. Nam , A. J. Giaccia , Trends Cancer 2016, 2, 295. 28741527 10.1016/j.trecan.2016.05.006 PMC5808868

[16] D. M. Gilkes , J. Biol. Chem. 2013, 288, 10819. 23423382 10.1074/jbc.M112.442939 PMC3624462

[17] M. Z. Noman , G. Desantis , B. Janji , M. Hasmim , S. Karray , P. Dessen , V. Bronte , S. Chouaib , J. Exp. Med. 2014, 211, 781. 24778419 10.1084/jem.20131916 PMC4010891

[18] S. M. Hatfield , J. Kjaergaard , D. Lukashev , T. H. Schreiber , B. Belikoff , R. Abbott , S. Sethumadhavan , P. Philbrook , K. Ko , R. Cannici , M. Thayer , S. Rodig , J. L. Kutok , E. K. Jackson , B. Karger , E. R. Podack , A. Ohta , M. V. Sitkovsky , Sci. Transl. Med. 2015, 7, 277ra30. 10.1126/scitranslmed.aaa1260 PMC4641038 25739764

[19] P. Jayaprakash , M. Ai , A. Liu , P. Budhani , T. Bartkowiak , J. Sheng , C. Ager , C. Nicholas , A. R. Jaiswal , Y. Sun , K. Shah , S. Balasubramanyam , N. Li , G. Wang , J. Ning , A. Zal , T. Zal , M. A. Curran , J. Clin. Invest. 2018, 128, 5137. 30188869 10.1172/JCI96268 PMC6205399

[20] M. Binnewies , E. W. Roberts , K. Kersten , V. Chan , D. F. Fearon , M. Merad , L. M. Coussens , D. I. Gabrilovich , S. Ostrand‐Rosenberg , C. C. Hedrick , R. H. Vonderheide , M. J. Pittet , R. K. Jain , W. Zou , T. K. Howcroft , E. C. Woodhouse , R. A. Weinberg , M. F. Krummel , Nat. Med. 2018, 24, 541. 29686425 10.1038/s41591-018-0014-x PMC5998822

[21] S. M. Hatfield , M. V. Sitkovsky , J. Clin. Invest. 2020, 130, 5629. 32870821 10.1172/JCI137554 PMC7598059

[22] W. Wang , Y. Cheng , P. Yu , H. Wang , Y. Zhang , H. Xu , Q. Ye , A. Yuan , Y. Hu , J. Wu , Nat. Commun. 2019, 10, 1580. 30952842 10.1038/s41467-019-09389-2 PMC6450981

[23] Z. Chen , L. Liu , R. Liang , Z. Luo , H. He , Z. Wu , H. Tian , M. Zheng , Y. Ma , L. Cai , ACS Nano 2018, 12, 8633. 30005164 10.1021/acsnano.8b04371

[24] Z. Zhou , B. Zhang , W. Zai , L. Kang , A. Yuan , Y. Hu , J. Wu , Proc. Natl. Acad. Sci. USA 2019, 116, 11972. 31142648 10.1073/pnas.1901987116 PMC6575596

[25] Y. Chao , L. Xu , C. Liang , L. Feng , J. Xu , Z. Dong , L. Tian , X. Yi , K. Yang , Z. Liu , Nat. Biomed. Eng. 2018, 2, 611. 31015634 10.1038/s41551-018-0262-6

[26] Y. Huang , J. Yuan , E. Righi , W. S. Kamoun , M. Ancukiewicz , J. Nezivar , M. Santosuosso , J. D. Martin , M. R. Martin , F. Vianello , P. Leblanc , L. L. Munn , P. Huang , D. G. Duda , D. Fukumura , R. K. Jain , M. C. Poznansky , Proc. Natl. Acad. Sci. USA 2012, 109, 17561. 23045683 10.1073/pnas.1215397109 PMC3491458

[27] N. E. Scharping , A. V. Menk , R. D. Whetstone , X. Zeng , G. M. Delgoffe , Cancer Immunol. Res. 2017, 5, 9. 27941003 10.1158/2326-6066.CIR-16-0103 PMC5340074

[28] J. Daruwalla , C. Christophi , World J. Surg. 2006, 30, 2112. 17102915 10.1007/s00268-006-0190-6

[29] H. Bitterman , Crit. Care 2009, 13, 205. 19291278 10.1186/cc7151 PMC2688103

[30] X. Wu , Y. Zhu , W. Huang , J. Li , B. Zhang , Z. Li , X. Yang , Adv. Sci. 2018, 5, 1700859. 10.1002/advs.201700859 PMC6097095 30128223

[31] J. Li , J. Huang , Y. Ao , S. Li , Y. Miao , Z. Yu , L. Zhu , X. Lan , Y. Zhu , Y. Zhang , X. Yang , ACS Appl. Mater. Interfaces 2018, 10, 22985. 29877702 10.1021/acsami.8b07090

[32] T. Stylianopoulos , J. D. Martin , V. P. Chauhan , S. R. Jain , B. Diop‐Frimpong , N. Bardeesy , B. L. Smith , C. R. Ferrone , F. J. Hornicek , Y. Boucher , L. L. Munn , R. K. Jain , Proc. Natl Acad. Sci. USA 2012, 109, 15101. 22932871 10.1073/pnas.1213353109 PMC3458380

[33] T. Stylianopoulos , J. D. Martin , M. Snuderl , F. Mpekris , S. R. Jain , R. K. Jain , Cancer Res. 2013, 73, 3833. 23633490 10.1158/0008-5472.CAN-12-4521 PMC3702668

[34] Q. Chen , J. Chen , Z. Yang , J. Xu , L. Xu , C. Liang , X. Han , Z. Liu , Adv. Mater. 2019, 31, 1802228. 10.1002/adma.201802228 30663118

[35] M. Overchuk , K. M. Harmatys , S. Sindhwani , M. A. Rajora , A. Koebel , D. M. Charron , A. M. Syed , J. Chen , M. G. Pomper , B. C. Wilson , W. C. W. Chan , G. Zheng , Nano Lett. 2021, 21, 344. 33301689 10.1021/acs.nanolett.0c03731

[36] S. R. Thom , J. Appl. Physiol. 2009, 106, 988. 18845776 10.1152/japplphysiol.91004.2008 PMC2660252

[37] K. Kessenbrock , V. Plaks , Z. Werb , Cell 2010, 141, 52. 20371345 10.1016/j.cell.2010.03.015 PMC2862057

[38] Z. Li , Y. Wang , Y. Shen , C. Qian , D. Oupicky , M. Sun , Sci. Adv. 2020, 6, eaaz9240. 32440550 10.1126/sciadv.aaz9240 PMC7228744

[39] R. Derynck , S. J. Turley , R. J. Akhurst , Nat. Rev. Clin. Oncol. 2021, 18, 9. 32710082 10.1038/s41571-020-0403-1 PMC9721352

[40] P. M. Tibbles , J. S. Edelsberg , N. Engl. J. Med. 1996, 334, 1642. 8628361 10.1056/NEJM199606203342506

[41] J. Cha , W. Yang , W. Xia , Y. Wei , L. Chan , S. Lim , C. Li , T. Kim , S. Chang , H. Lee , J. L. Hsu , H. Wang , C. Kuo , W. Chang , S. Hadad , C. A. Purdie , A. M. McCoy , S. Cai , Y. Tu , J. K. Litton , E. A. Mittendorf , S. L. Moulder , W. F. Symmans , A. M. Thompson , H. Piwnica‐Worms , C. Chen , K. Khoo , M. Hung , Mol. Cell 2018, 71, 606. 30118680 10.1016/j.molcel.2018.07.030 PMC6786495

[42] K. Newick , S. O'Brien , E. Moon , S. M. Albelda , Rev. Med. 2017, 68, 139. 10.1146/annurev-med-062315-120245 27860544

[43] Q. Chen , Q. Hu , E. Dukhovlinova , G. Chen , S. Ahn , C. Wang , E. A. Ogunnaike , F. S. Ligler , G. Dotti , Z. Gu , Adv. Mater. 2019, 31, 1900192. 10.1002/adma.201900192 PMC7262962 30916367

[44] I. Caruana , B. Savoldo , V. Hoyos , G. Weber , H. Liu , E. S. Kim , M. M. Ittmann , D. Marchetti , G. Dotti , Nat. Med. 2015, 21, 524. 25849134 10.1038/nm.3833 PMC4425589

[45] M. J. Ware , V. Keshishian , J. J. Law , J. C. Ho , C. A. Favela , P. Rees , B. Smith , S. Mohammad , R. F. Hwang , K. Rajapakshe , C. Coarfa , S. Huang , D. P. Edwards , S. J. Corr , B. Godin , S. A. Curley , Biomaterials 2016, 108, 129. 27627810 10.1016/j.biomaterials.2016.08.041 PMC5082237

