# Supplementary material for: Hyperbaric Oxygen Boosts PD‐1 Antibody Delivery and T Cell Infiltration for Augmented Immune Responses Against Solid Tumors
Source: Adv Sci (Weinh). 2021 Jun 3;8(15):2100233. doi: 10.1002/advs.202100233 (PMC8336507; doi:10.1002/advs.202100233)
Supplement: Supplementary file 1 — Supporting Information [file ADVS-8-2100233-s001.pdf]

© 2021 Wiley-VCH GmbH

ADVANCED SCIENCE
Open Access

Supporting Information

for *Adv. Sci.*, DOI: 10.1002/advs.202100233

Hyperbaric oxygen boosts PD-1 antibody delivery and T cell infiltration for augmented immune responses against solid tumors

Xin Liu, Ningbing Ye, Sha Liu, Jiankun Guan, Qingyuan Deng, Zhijie Zhang, Chen Xiao, Ze-yang Ding, Bi-xiang Zhang, Xiao-ping Chen, Zifu Li, and Xiangliang Yang*

Supplementary Materials for

Hyperbaric oxygen boosts PD-1 antibody delivery and T cell infiltration for augmented immune responses against solid tumors

Xin Liu, Ningbing Ye, Sha Liu, Jiankun Guan, Qingyuan Deng, Zhijie Zhang, Chen Xiao, Ze-yang Ding, Bi-xiang Zhang, Xiao-ping Chen, Zifu Li, and Xiangliang Yang*

*Corresponding author. E-mail: zifuli@hust.edu.cn

This PDF file includes:

Figure S1. PD-L1 expression study.
Figure S2. Tumor images of H22 orthotopic tumor model.
Figure S3. Tumor images and tumor metastasis evaluation of Panc02 orthotopic tumor model.
Figure S4. Tumor images and tumor metastasis evaluation of 4T1 orthotopic tumor model.
Figure S5. Antitumor effect of HBO and PD-1 Ab combination therapy in H22 subcutaneous tumor model.
Figure S6. Antitumor effect of HBO and PD-1 Ab combination therapy in H22 subcutaneous terminal tumor model.
Figure S7. Antitumor efficacy evaluation by tumor sections staining of H22 orthotopic tumor-bearing mice.
Figure S8. Antitumor efficacy evaluation by tumor sections staining of Panc02 orthotopic tumor-bearing mice.
Figure S9. Antitumor efficacy evaluation by tumor sections staining of 4T1 orthotopic tumor-bearing mice.
Figure S10. Antitumor efficacy evaluation by tumor sections staining of H22 subcutaneous tumor-bearing mice.
Figure S11. In vivo biocompatibility evaluation of H22 orthotopic tumor model.
Figure S12. In vivo biocompatibility evaluation of Panc02 orthotopic tumor model.

Figure S13. In vivo biocompatibility evaluation of 4T1 orthotopic tumor model.

Figure S14. In vivo biocompatibility evaluation of H22 subcutaneous tumor model.

Figure S15. Transcriptome analysis of regulating TME by HBO.

Figure S16. Heat map analysis of the major regulated cell components and signaling pathway after HBO treatment.

Figure S17. HBO modulates mechanical TME.

Figure S18. Image of tumors used for solid stress quantification.

Figure S19. Hydrodynamic diameter of PD-1 Ab measured by DLS.

Figure S20. HBO down-regulates the expression of PD-L1.

Figure S21. HBO reduces MDSCs and M2 TAM in tumor tissues.

Figure.S22. HBO slightly decreases Treg in tumor tissues.

Figure S23. HBO regulates cytokines in serum and tumor tissues.

Figure S24. HBO modulates enzyme activity in tumor tissues.

Figure S25. Clustering diagram of flow cytometry analysis in immune TME evaluation.

Figure S26. Tumor images of relapse H22 tumor model.

Figure S27. CTL proportion analysis in relapse H22 tumor model.

Figure S28. Clustering diagram of flow cytometry analysis in relapse H22 tumor model.

Figure S29. Clinical tumor samples images of different patients used in this study.

Figure S30. HBO increases MMP-7 expression in tumor tissues.

Table S1. Patient demographics.

Supplementary Figures

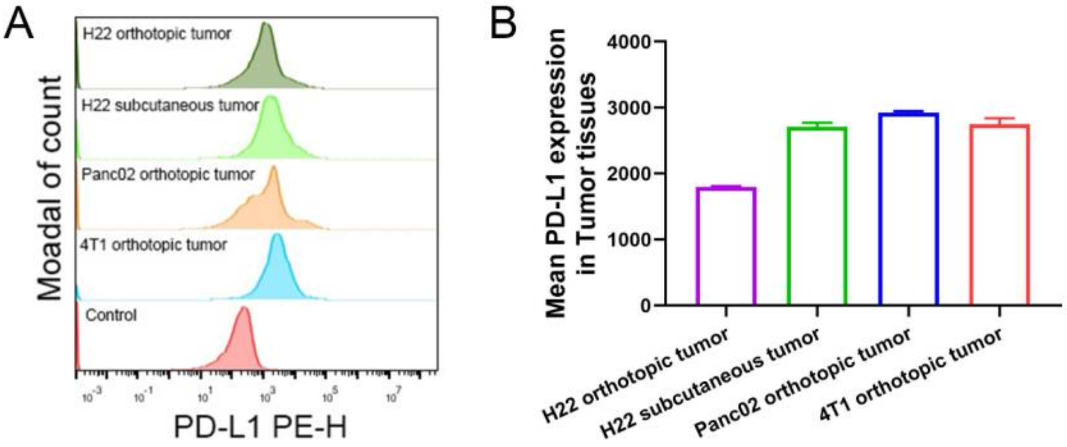

Figure S1. PD-L1 expression study. (A) Flow cytometry analysis of the PD-L1 expression in tissues of H22 orthotopic tumor, H22 subcutaneous tumor, Panc02 orthotopic tumor and 4T1 orthotopic tumor. (B) Mean PD-L1 expression in four types of tumor tissues. Error bars indicate SEM (n=3).

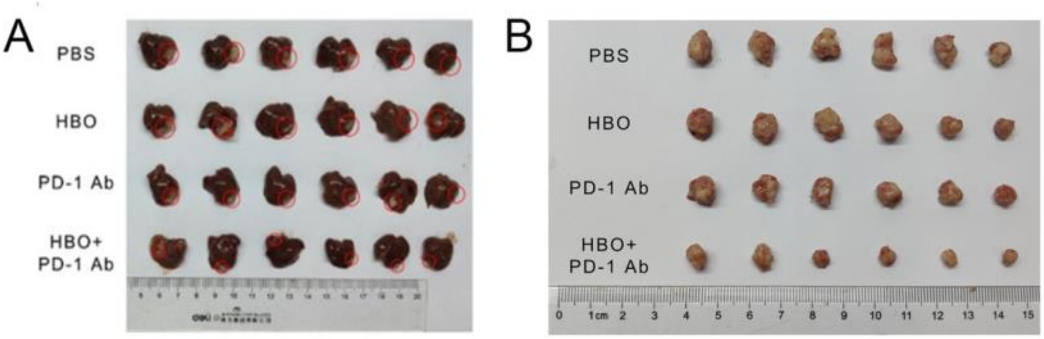

Figure S2. Tumor images of H22 orthotopic tumor model. Liver (A) and tumor (B) images of H22 orthotopic tumor-bearing BALB/c mice after different treatments (n=6).

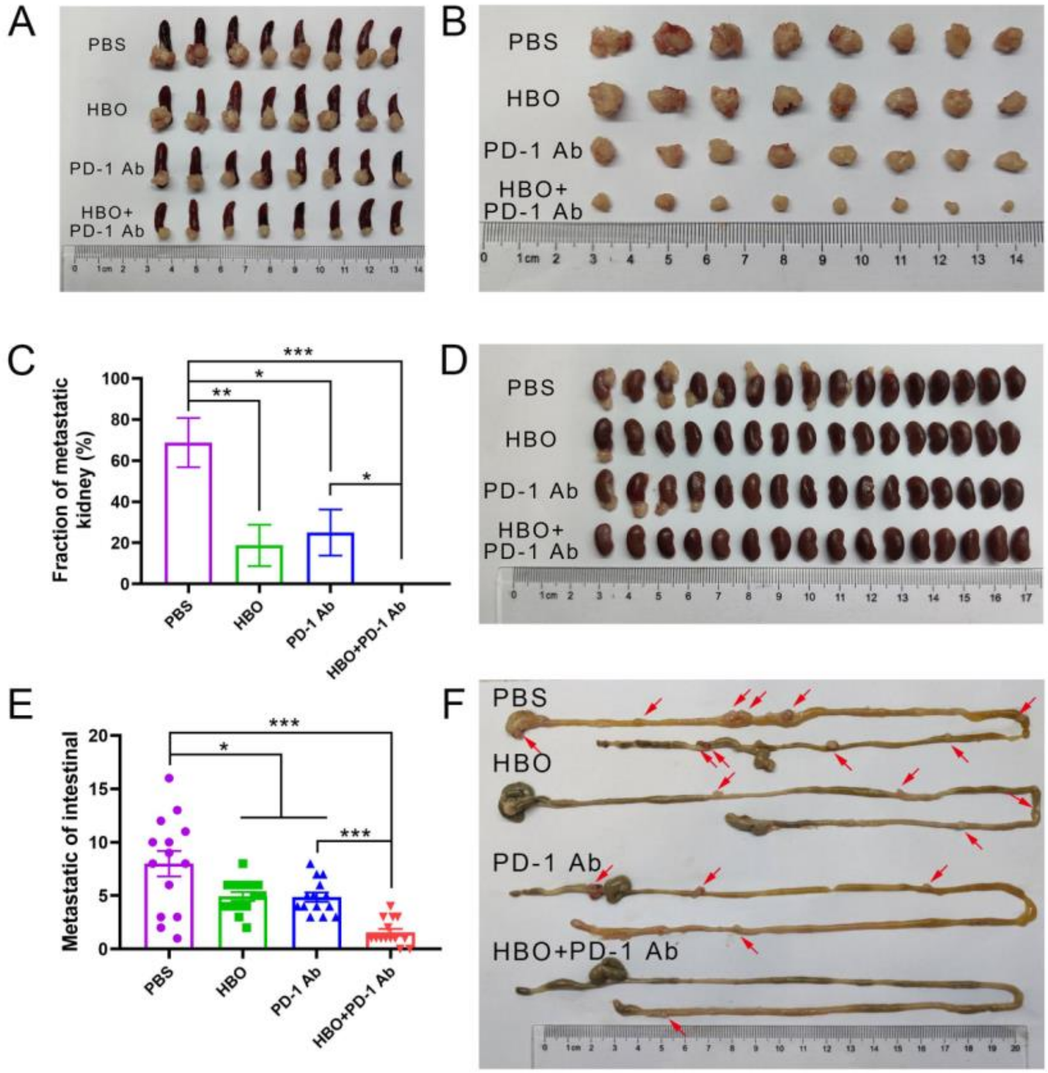

Figure S3. Tumor images and tumor metastasis evaluation of Panc02 orthotopic tumor model. Spleen (A) and tumor (B) images of Panc02 orthotopic tumor-bearing BALB/c mice after different treatments. (C, D) Fraction of metastatic Kidney. (E, F) Metastatic nodules in mouse intestinal. Error bars indicate SEM (n=8). Statistical significance was calculated by t-test. P-values: *, $P < 0.05$; **, $P < 0.01$; ***, $P < 0.001$.

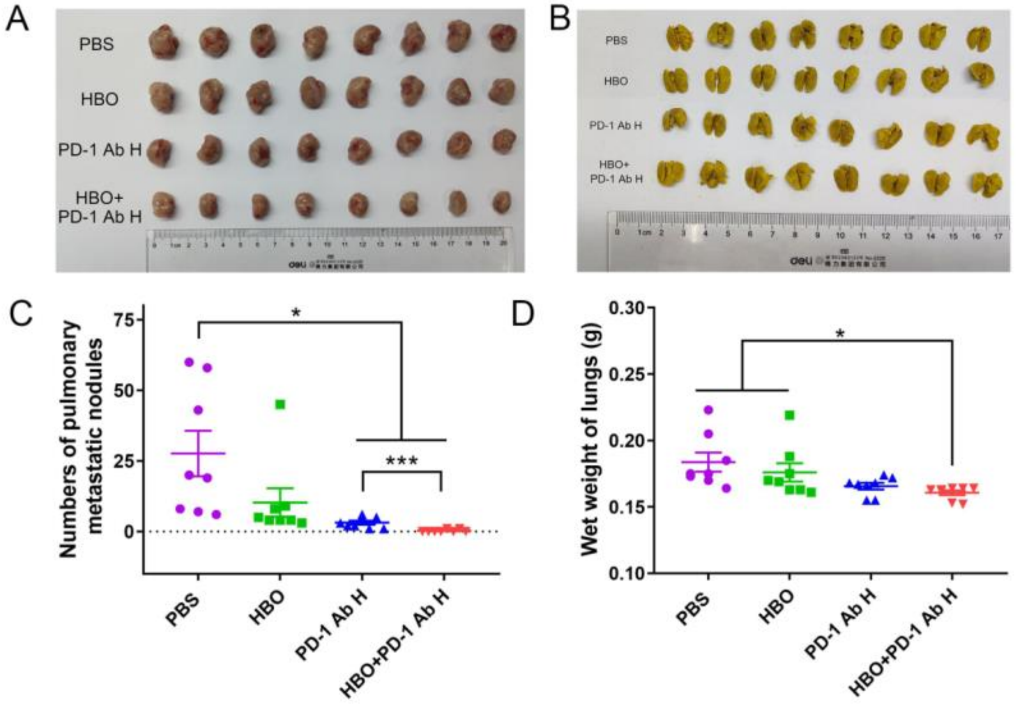

Figure S4. Tumor images and tumor metastasis evaluation of 4T1 orthotopic tumor model. Tumor (A) and lung (B) images of 4T1 orthotopic tumor-bearing BALB/c mice after different treatments. (C) Numbers of pulmonary metastatic nodules in Lungs. (D) Wet weight of lung of orthotopic tumor-bearing BALB/c mice. Error bars indicate SEM (n=8). Statistical significance was calculated by t-test. P-values: *, P < 0.05; **, P < 0.01; ***, P < 0.001.

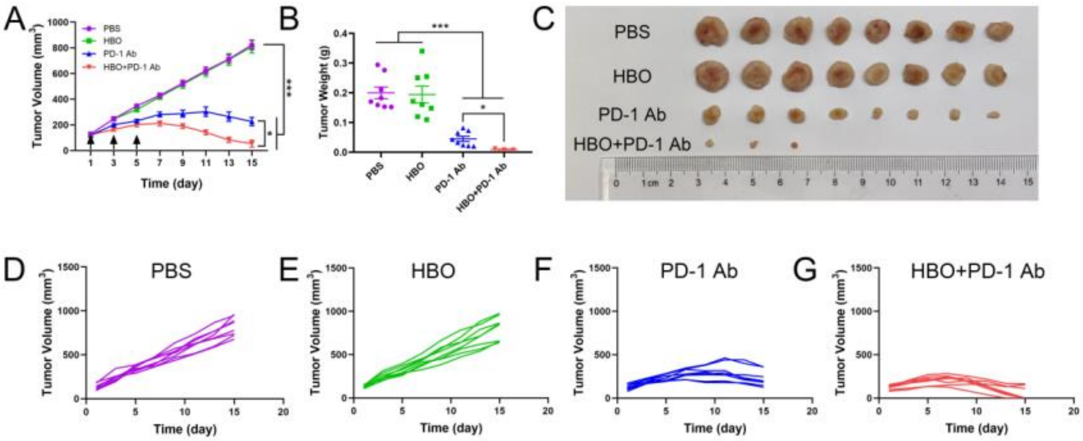

Figure S5. Antitumor effect of HBO and PD-1 Ab combination therapy in H22 subcutaneous tumor model. Tumor growth curves (A), tumor weight (B) and tumor image of H22 subcutaneous tumor-bearing BALB/c mice after different treatments. Tumor growth curve of single mouse of PBS

group (D), HBO (E), PD-1 Ab (F), and HBO+PD-1 Ab group (G) of H22 subcutaneous tumor-bearing BALB/c mice. Error bars indicate SEM (n=8). Statistical significance was calculated by t-test. P-values: *, P < 0.05; **, P < 0.01; ***, P < 0.001.

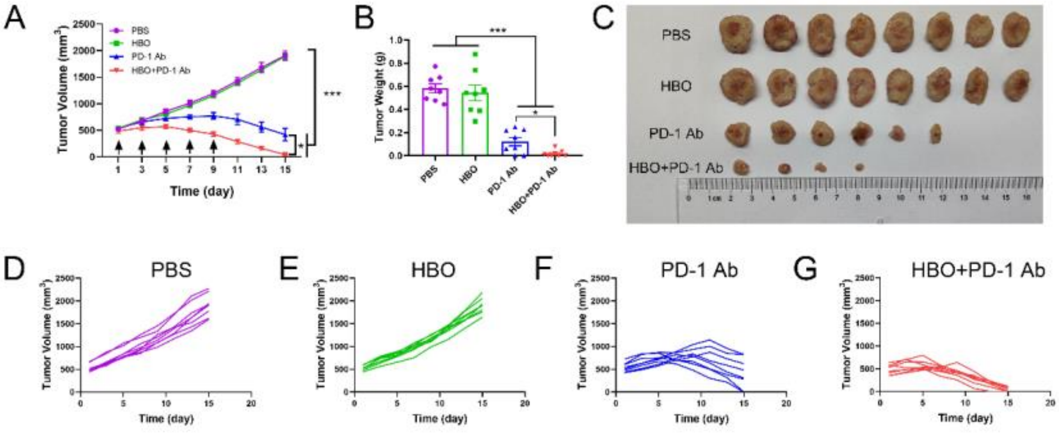

Figure S6. Antitumor effect of HBO and PD-1 Ab combination therapy in H22 subcutaneous terminal tumor model. Tumor growth curves (A), tumor weight (B) and tumor image (C) of H22 subcutaneous tumor-bearing BALB/c mice after different treatments. Tumor growth curve of single mouse of PBS group (D), HBO (E), PD-1 Ab (F), and HBO+PD-1 Ab group (G) of H22 subcutaneous tumor-bearing BALB/c mice. Error bars indicate SEM (n=8). Statistical significance was calculated by t-test. P-values: *, P < 0.05; **, P < 0.01; ***, P < 0.001.

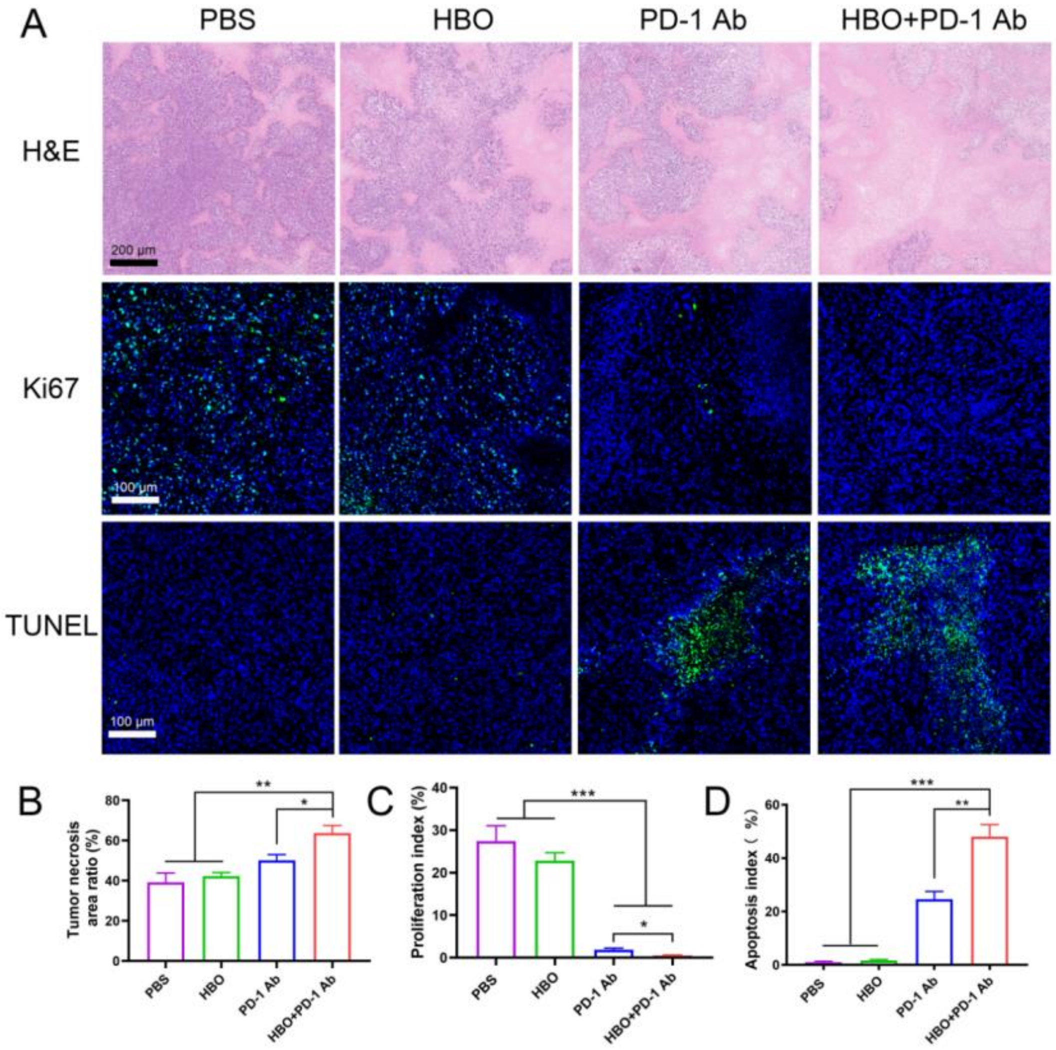

Figure S7. Antitumor efficacy evaluation by tumor sections staining of H22 orthotopic tumor-bearing mice. (A) H&E staining, ki67 immunofluorescence staining, and TUNEL immunofluorescence staining of tumor sections at the end of the in vivo H22 orthotopic tumor model experiment. (B) Semi-quantification of necrosis rate of the tumor tissue in the H&E staining assay. (C) Semi-quantification of proliferation index of the tumor tissue in the Ki67 staining assay. (D) Semi-quantification of apoptosis index of the tumor tissue in the TUNEL staining. Error bars indicate SEM (n=3). Statistical significance was calculated by t-test. P-values: *, $P < 0.05$; **, $P < 0.01$; ***, $P < 0.001$.

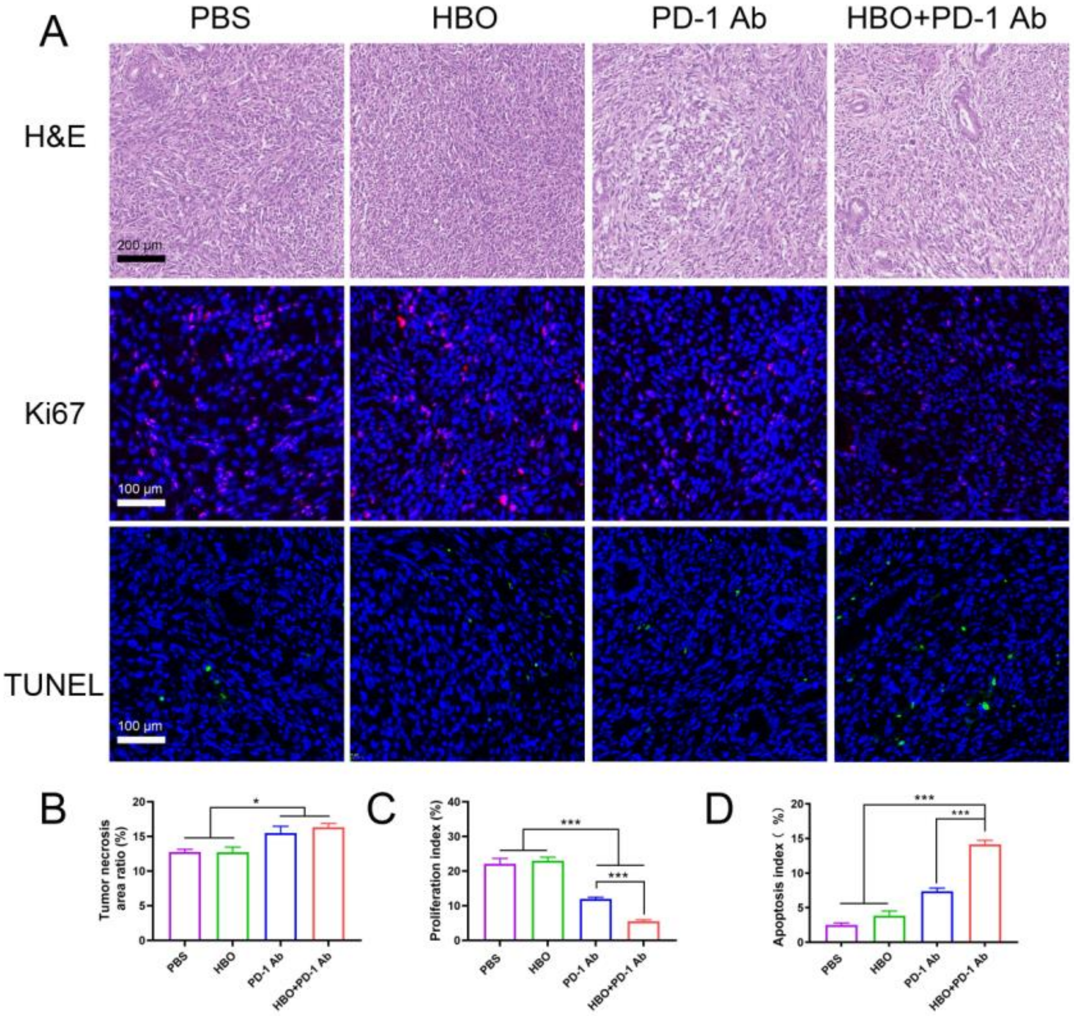

Figure S8. Antitumor efficacy evaluation by tumor sections staining of Panc02 orthotopic tumor-bearing mice. (A) H&E staining, ki67 immunofluorescence staining, and TUNEL immunofluorescence staining of tumor sections at the end of the in vivo Panc02 orthotopic tumor model experiment. (B) Semi-quantification of necrosis rate of the tumor tissue in the H&E staining assay. (C) Semi-quantification of proliferation index of the tumor tissue in the Ki67 staining assay. (D) Semi-quantification of apoptosis index of the tumor tissue in the TUNEL staining. Error bars indicate SEM (n=3). Statistical significance was calculated by t-test. P-values: *, P < 0.05; **, P < 0.01; ***, P < 0.001.

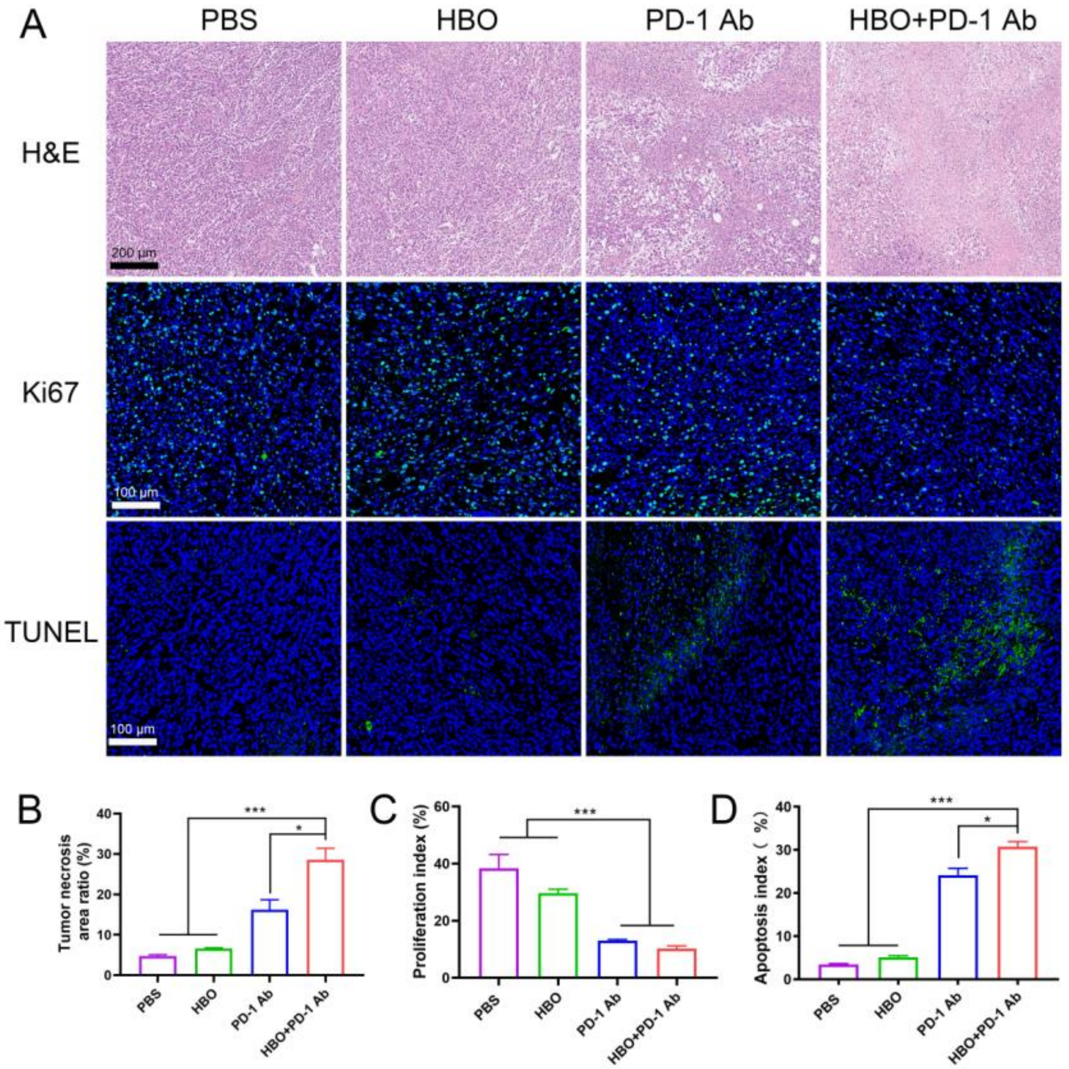

Figure S9. Antitumor efficacy evaluation by tumor sections staining of 4T1 orthotopic tumor-bearing mice. (A) H&E staining, ki67 immunofluorescence staining, and TUNEL immunofluorescence staining of tumor sections at the end of the in vivo 4T1 orthotopic tumor model experiment. (B) Semi-quantification of necrosis rate of the tumor tissue in the H&E staining assay. (C) Semi-quantification of proliferation index of the tumor tissue in the Ki67 staining assay. (D) Semi-quantification of apoptosis index of the tumor tissue in the TUNEL staining. Error bars indicate SEM (n=3). Statistical significance was calculated by t-test. P-values: *, $P < 0.05$; **, $P < 0.01$; ***, $P < 0.001$.

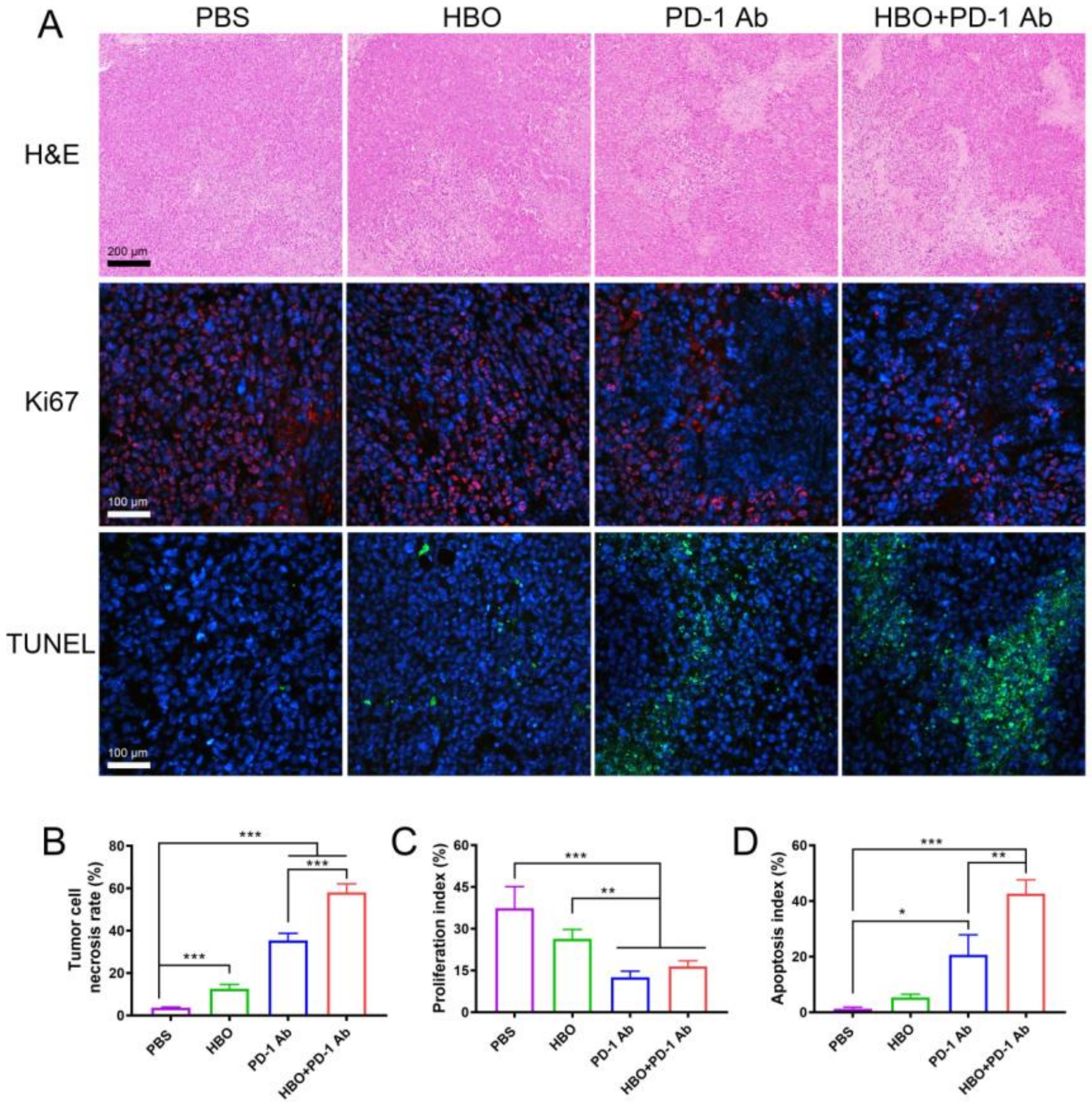

Figure S10. Antitumor efficacy evaluation by tumor sections staining of H22 subcutaneous tumor-bearing mice. (A) H&E staining, ki67 immunofluorescence staining, and TUNEL immunofluorescence staining of tumor sections at the end of the in vivo H22 subcutaneous tumor model experiment. (B) Semi-quantification of necrosis rate of the tumor tissue in the H&E staining assay. (C) Semi-quantification of proliferation index of the tumor tissue in the Ki67 staining assay. (D) Semi-quantification of apoptosis index of the tumor tissue in the TUNEL staining. Error bars indicate SEM (n=3). Statistical significance was calculated by t-test. P-values: *, $P < 0.05$; **, $P < 0.01$; ***, $P < 0.001$.

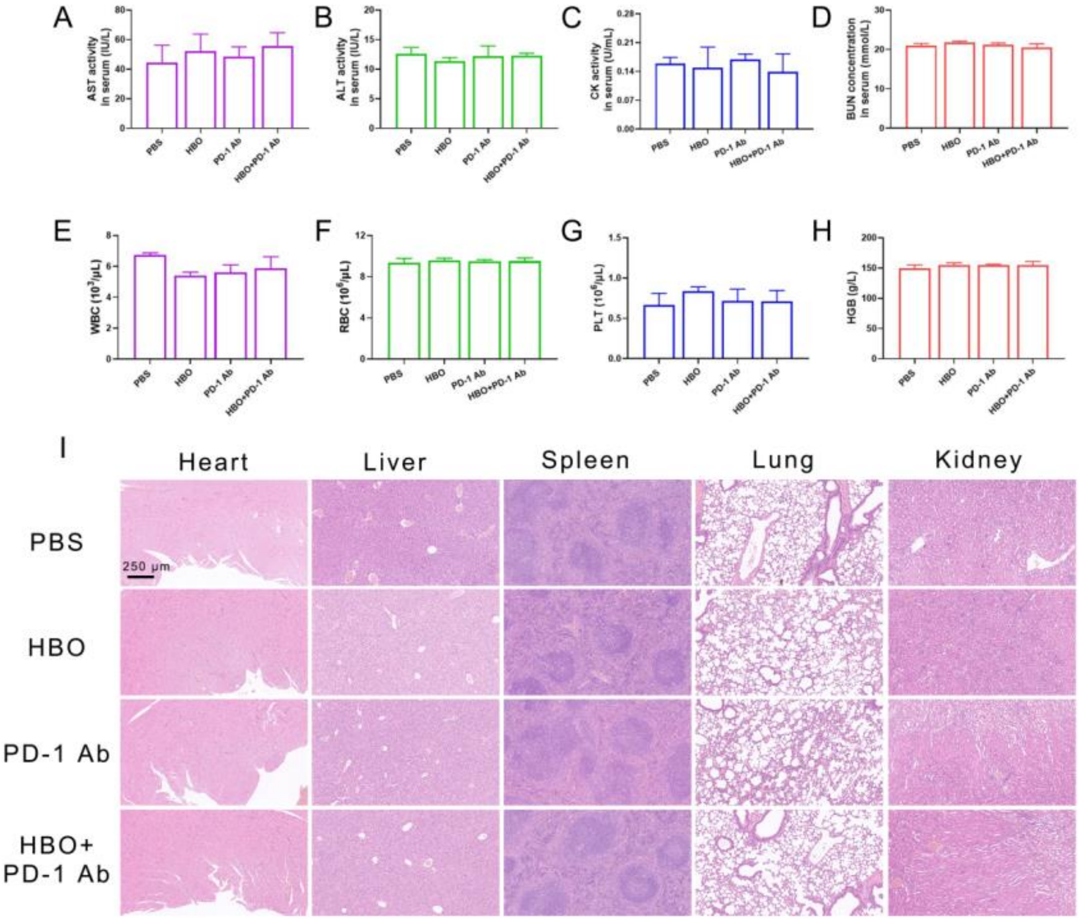

Figure S11. In vivo biocompatibility evaluation of H22 orthotopic tumor model. AST(A), ALT(B), CK (C) and BUN (D) activity in serum. (E) Numbers of white blood cell (WBC) in serum. (F) Numbers of red blood cell (RBC) in different groups. (G) Numbers of platelet in serum. (H) Concentration of HGB in serum. (I) Pathological analysis of mice major organs in different groups. Error bars indicate SEM (n=6).

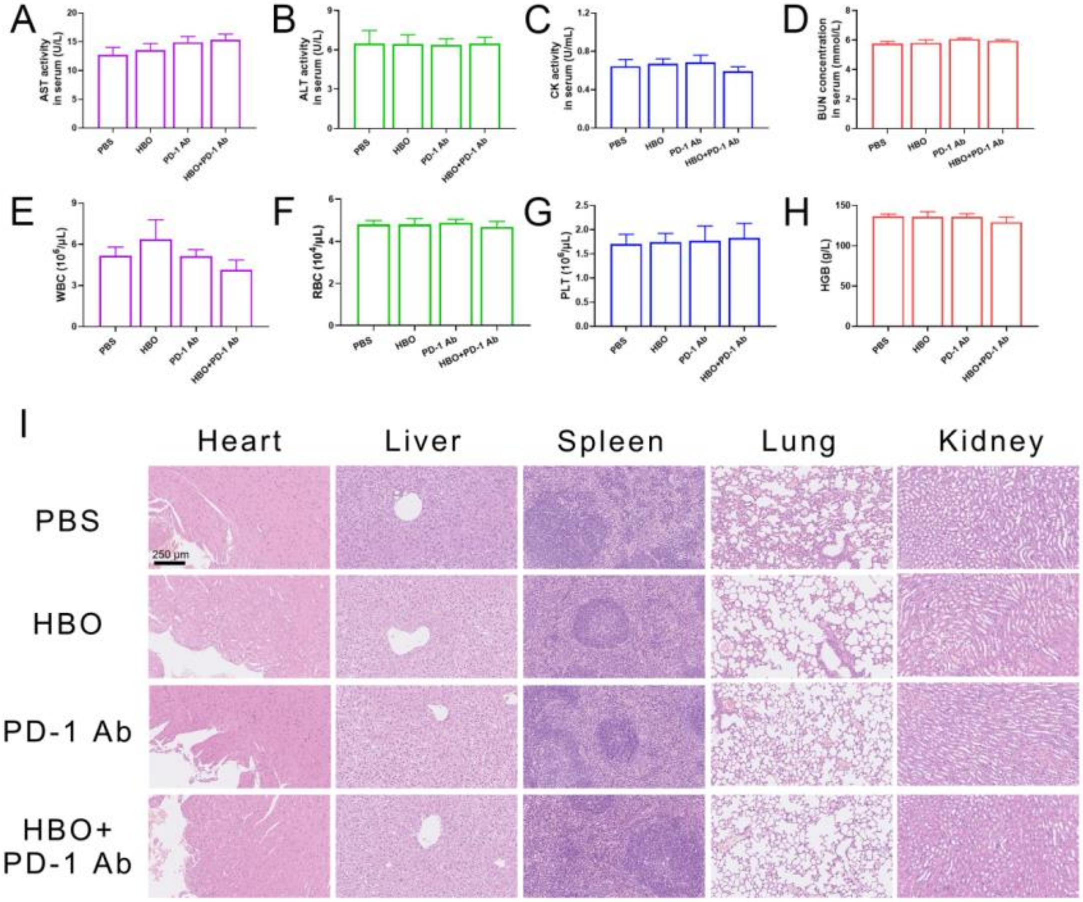

Figure S12. In vivo biocompatibility evaluation of Panc02 orthotopic tumor model. AST (A), ALT(B), CK (C) and BUN (D) activity in serum. (E) Numbers of white blood cell (WBC) in serum. (F) Numbers of red blood cell (RBC) in different groups. (G) Numbers of platelet in serum. (H) Concentration of HGB in serum. (I) Pathological analysis of mice major organs in different groups. Error bars indicate SEM (n=6).

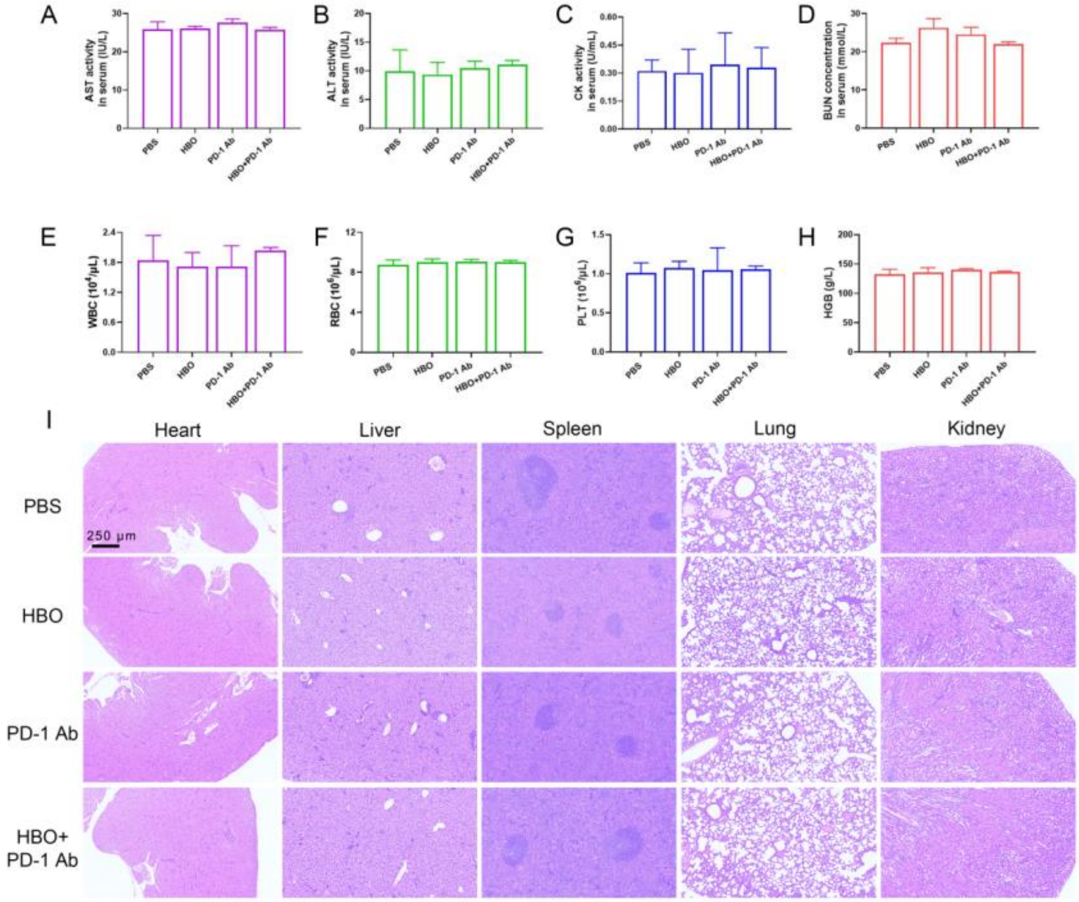

Figure S13. In vivo biocompatibility evaluation of 4T1 orthotopic tumor model. AST (A), ALT(B), CK (C) and BUN (D) activity in serum. (E) Numbers of white blood cell (WBC) in serum. (F) Numbers of red blood cell (RBC) in different groups. (G) Numbers of platelet in serum. (H) Concentration of HGB in serum. (I) Pathological analysis of mice major organs in different groups. Error bars indicate SEM (n=6).

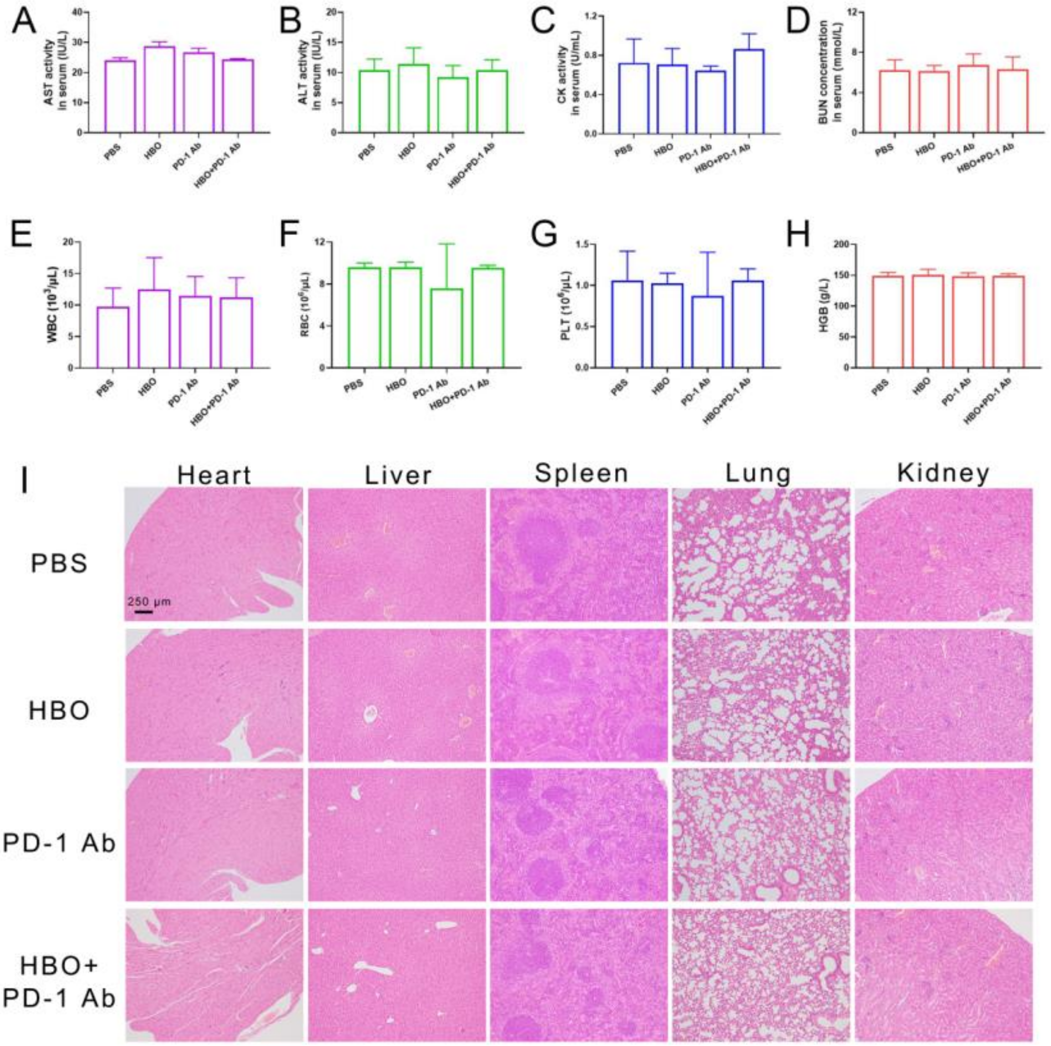

Figure S14. In vivo biocompatibility evaluation of H22 subcutaneous tumor model. AST (A), ALT(B), CK (C) and BUN (D) activity in serum. (E) Numbers of white blood cell (WBC) in serum. (F) Numbers of red blood cell (RBC) in different groups. (G) Numbers of platelet in serum. (H) Concentration of HGB in serum. (I) Pathological analysis of mice major organs in different groups. Error bars indicate SEM (n=6).

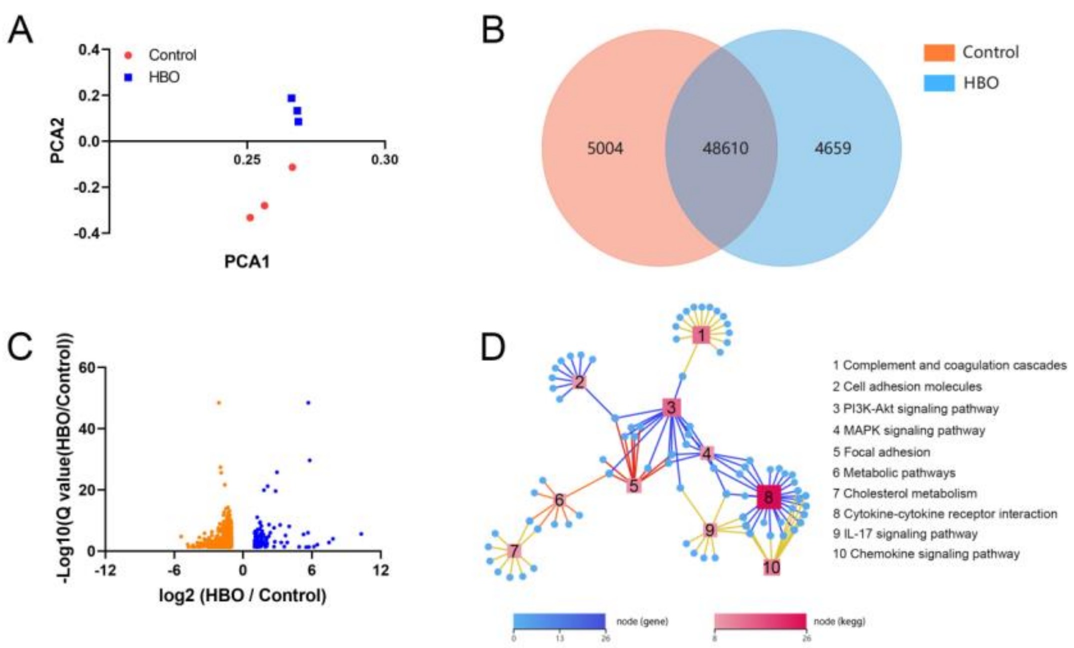

Figure S15. Transcriptome analysis of regulating TME by HBO. (A) Principal component analysis (PCA) of two groups, control and HBO. Each data point corresponds to the PCA analysis of each sample. (B) Venn diagram of the transcriptomic profiles between HBO and control groups. (C) Volcano plots showing the identified upregulated and downregulated genes by HBO treatment. (D) Integrated regulatory KEGG network significantly influenced by HBO (n=3).

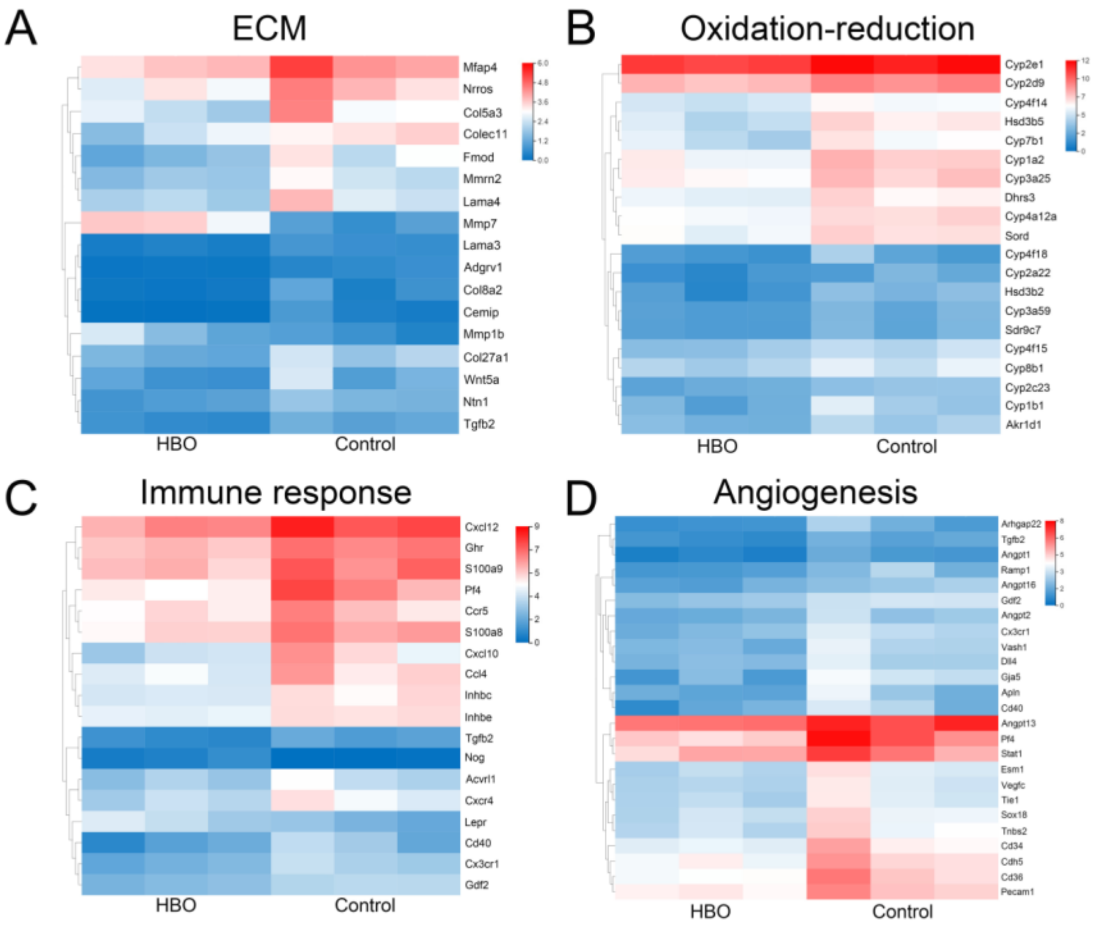

Figure S16. Heat map analysis of the major regulated cell components and signaling pathway after HBO treatment. Heat maps of significantly differentially expressed genes related to ECM (A), Oxidation-reduction (B), immune signaling pathways (C) and angiogenesis (D) (n=3).

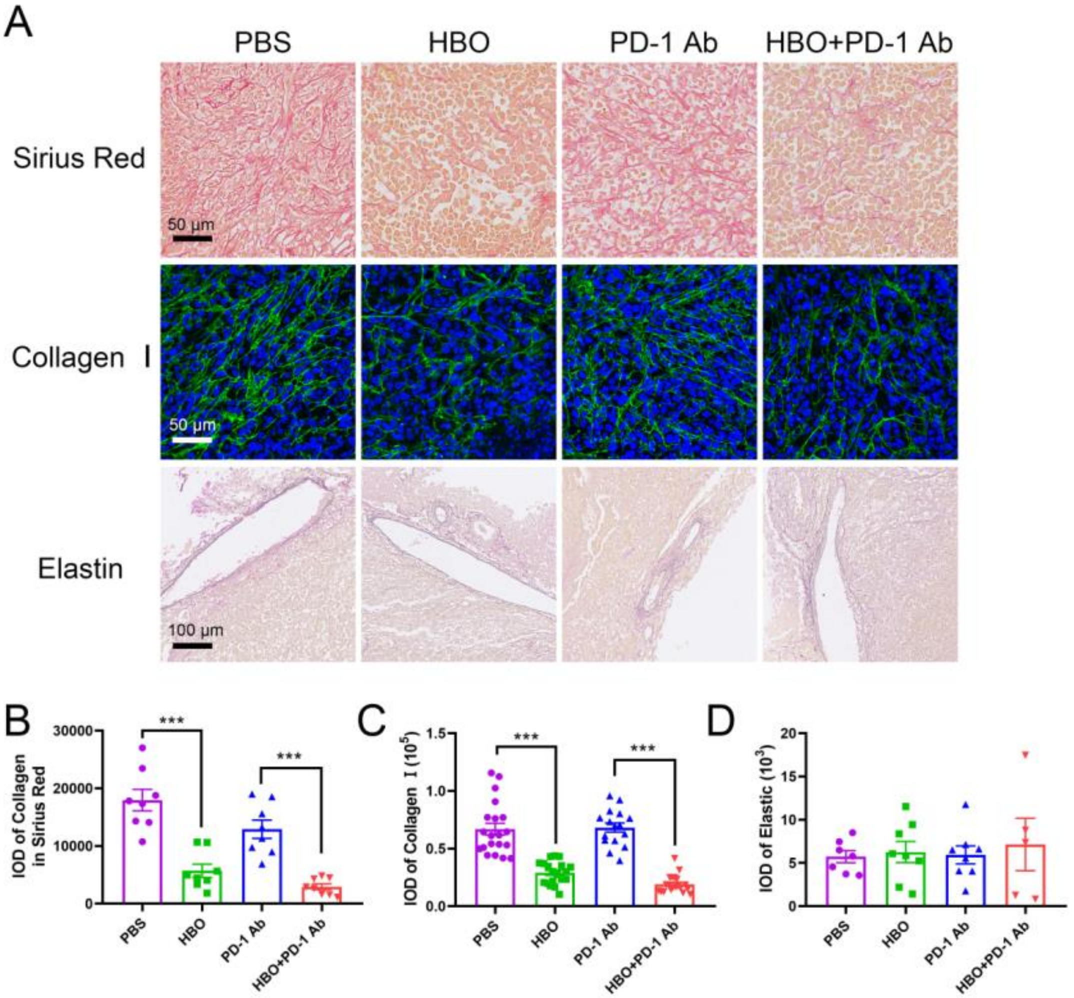

Figure S17. HBO modulates mechanical TME. (A) Representative Sirius Red staining images, immunofluorescence staining of collagen Ⅰ image and elastin image of tumor tissues. Quantification of collagen in Sirius Red staining (B), immunofluorescence staining (C) and elastin (D) in immunofluorescence staining of tumor tissue sections, respectively. Error bars indicate SEM (n=20). Statistical significance was calculated by t-test. P-values: *, $P < 0.05$; **, $P < 0.01$; ***, $P < 0.001$.

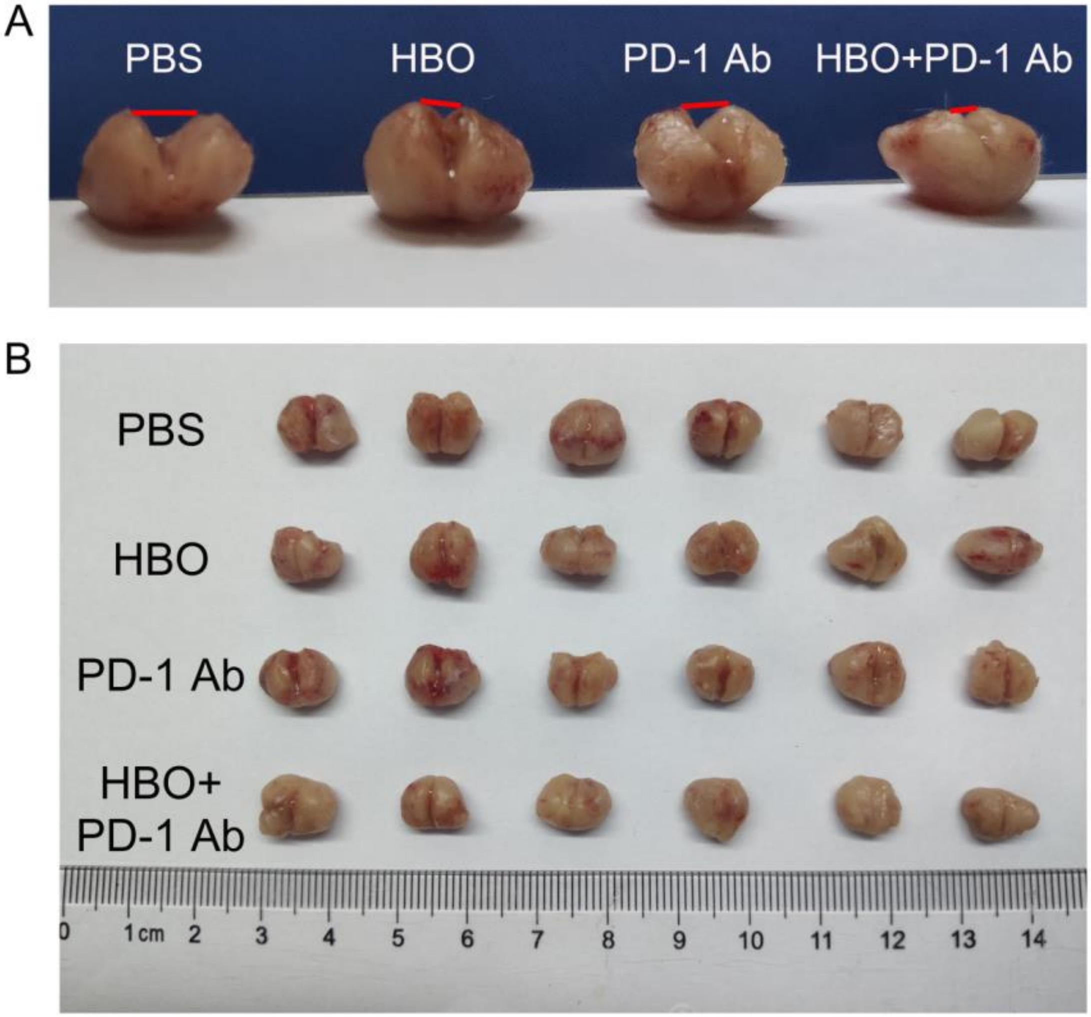

Figure S18. Image of tumors used for solid stress quantification. Flat view (A) and top view (B) photographs of 4T1 orthotopic tumors after making a cut to detected solid stress of tumor (n=6).

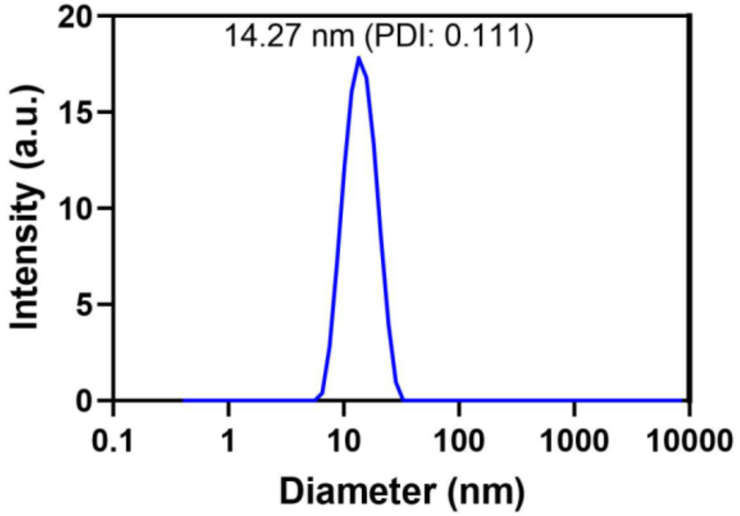

Figure S19. Hydrodynamic diameter of PD-1 Ab measured by DLS.

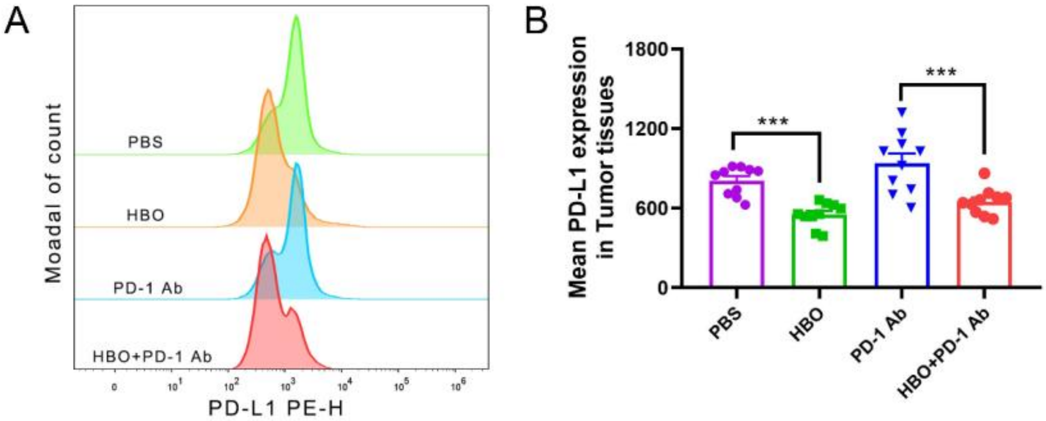

Figure S20. HBO down-regulates the expression of PD-L1. (A) Flow cytometry analysis of the PD-L1 expression in H22 orthotopic tumor. (B) Mean PD-L1 expression in H22 orthotopic tumor tissues. Error bars indicate SEM (n=10). Statistical significance was calculated by t-test. P-values: *, $P < 0.05$; **, $P < 0.01$; ***, $P < 0.001$.

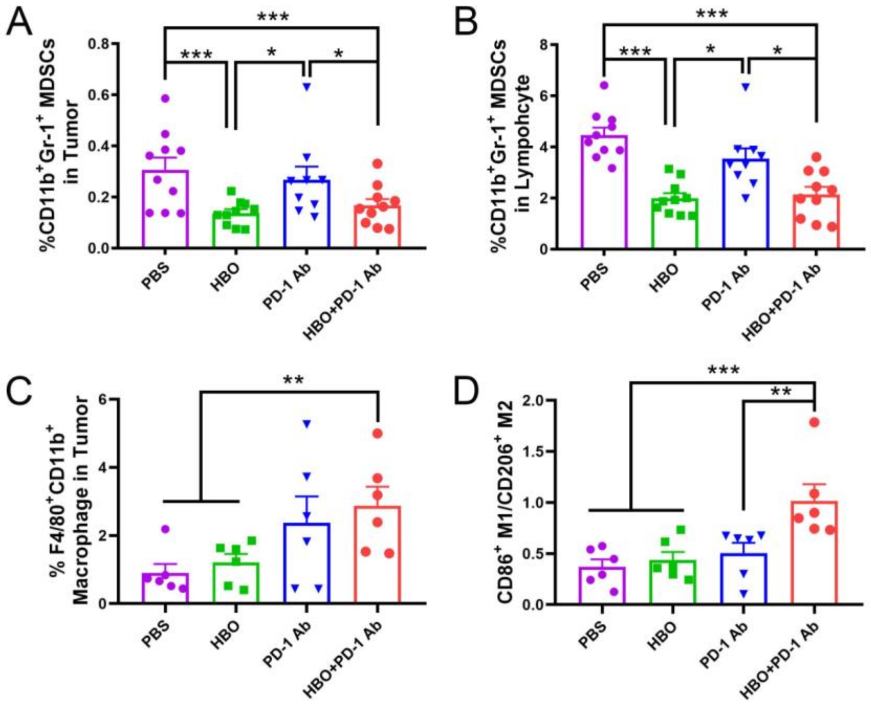

Figure S21. **HBO reduces MDSCs and M2 TAM in tumor tissues**. Proportion of infiltrated MDSCs gated on tumor cells (A) and gated on lymphocyte (B) in H22 orthotopic tumor after various treatments analyzed by flow cytometry (n=10). (C) Proportion of infiltrated tumor associated macrophages (gated on tumor cells) and the ratio of M1 to M2 (D) in H22 orthotopic tumor analyzed by flow cytometry (n=6). Error bars indicate SEM. Statistical significance was calculated by t-test. P-values: *, P < 0.05; **, P < 0.01; ***, P < 0.001.

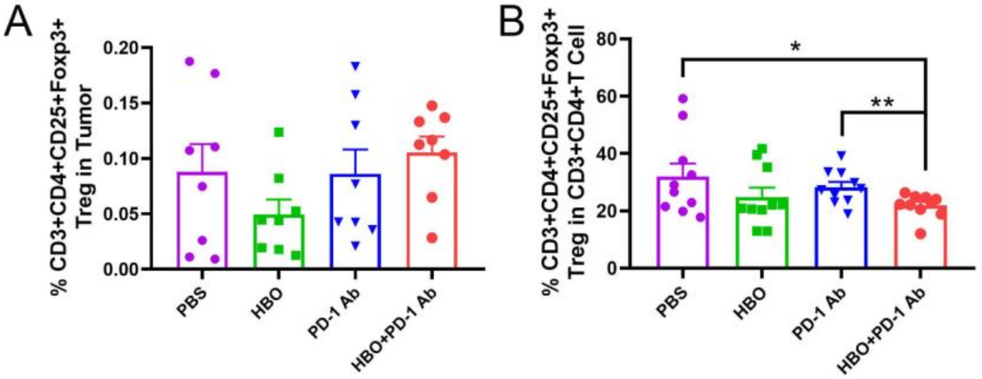

Figure S22. **HBO slightly decreases T_{reg} in tumor tissues.** Proportion of infiltrated T_{reg} gated on tumor cells (A) and gated on $CD4^{+}$ T cells (B) in H22 orthotopic tumor after various treatments analyzed by flow cytometry. Error bars indicate SEM (n=8). Statistical significance was calculated by t-test. P-values: *, P < 0.05; **, P < 0.01; ***, P < 0.001.

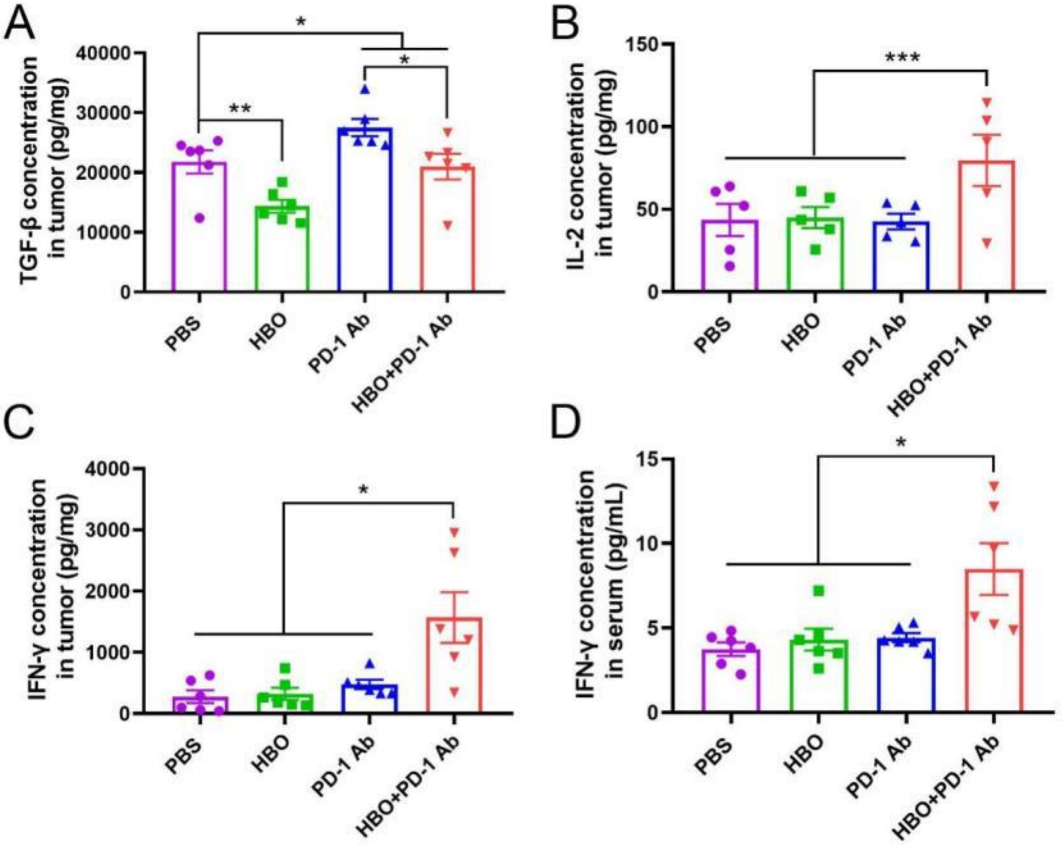

Figure S23. **HBO regulates cytokines in serum and tumor tissues.** Cytokine of TGF-β (A), IL-2(B), IFN-γ (C) in tumor, IFN-γ (D) in serum from H22 orthotopic tumor-bearing mice analyzed by ELISA. Error bars indicate SEM (n=6). Statistical significance was calculated by t-test. P-values: *, P < 0.05; **, P < 0.01; ***, P < 0.001.

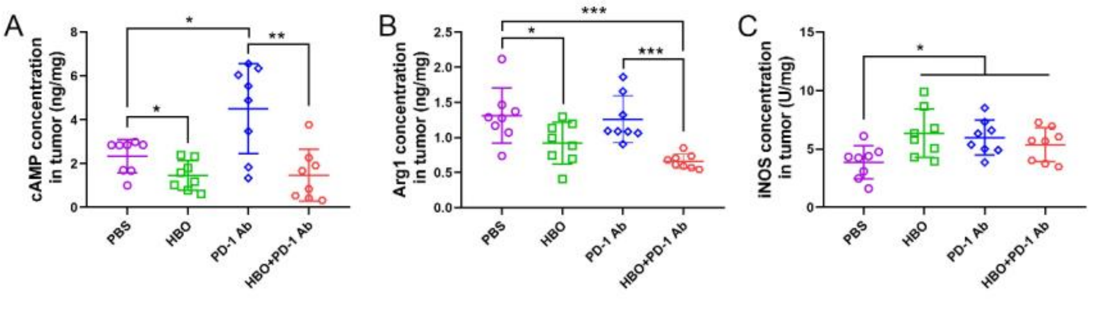

Figure S24. HBO modulates enzyme activity in tumor tissues. (A) cAMP, (B) Arg1 and (C) iNOS activity in H22 orthotopic tumor after treatments. Error bars indicate SEM (n=8). Statistical significance was calculated by t-test. P-values: *, P < 0.05; **, P < 0.01; ***, P < 0.001.

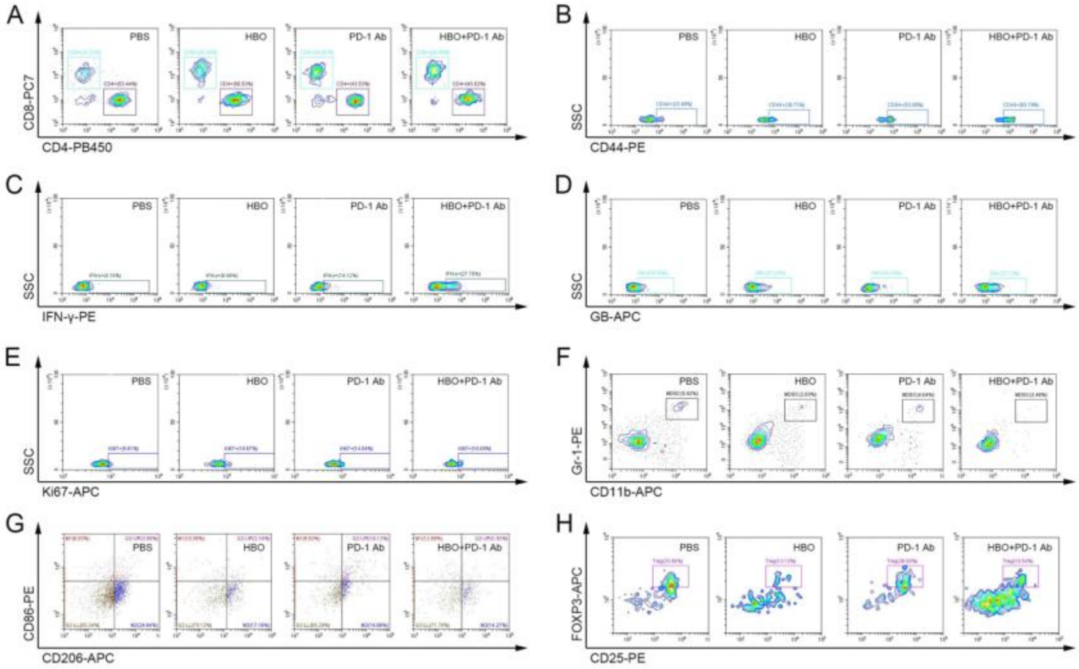

Figure S25. Clustering diagram of flow cytometry analysis in immune TME evaluation. Clustering diagram of CD4+/CD8+ (A), CD44+ (B), IFN-γ (C), granzyme B (D), Ki67 (E), MDSCs (F), TAMs (G) and Tregs (H) detected by flow cytometry in Fig. 4 and Fig. 5.

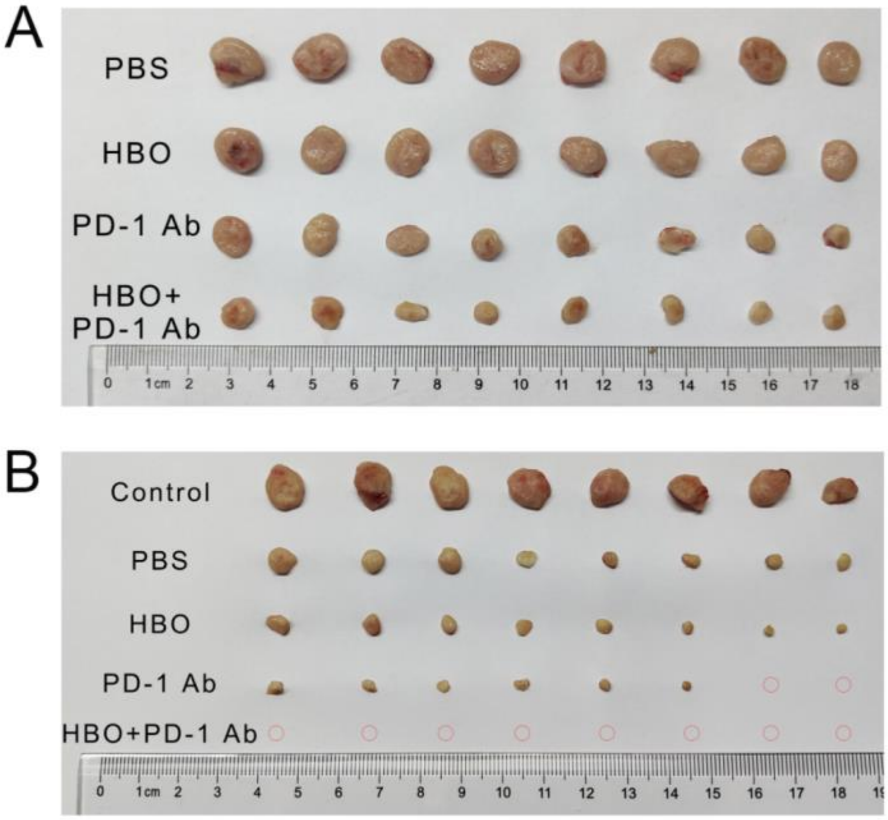

Figure S26. Tumor images of relapse H22 tumor model. Tumor images of the first tumors (A) and the rechallenged tumors (B) (n=8).

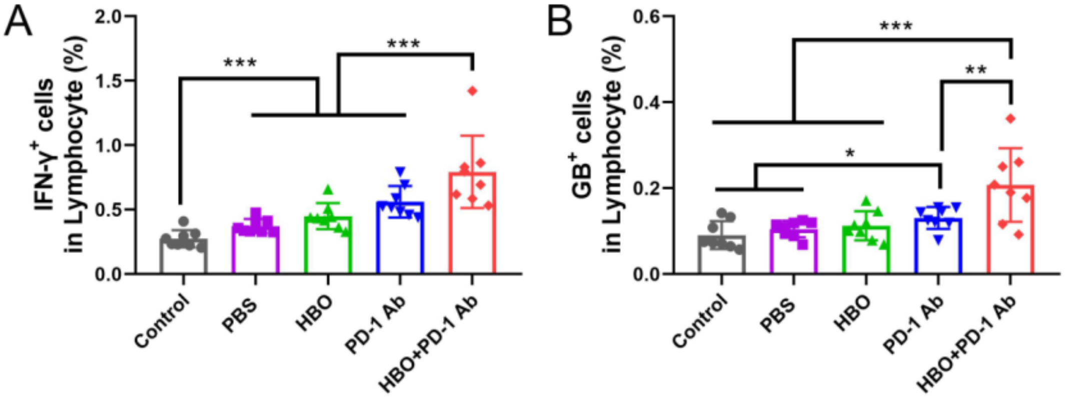

Figure S27. **CTL proportion analysis in relapse H22 tumor model.** Proportion of infiltrated IFN-γ (A) and granzyme B (B) expression of CD8+ T cells gated on lymphocyte in lymph node from mice isolated 10 days after mice were re-challenged with the secondary tumors analyzed by flow

cytometry. Error bars indicate SEM (n=8). Statistical significance was calculated by t-test. P-values: *, P < 0.05; **, P < 0.01; ***, P < 0.001.

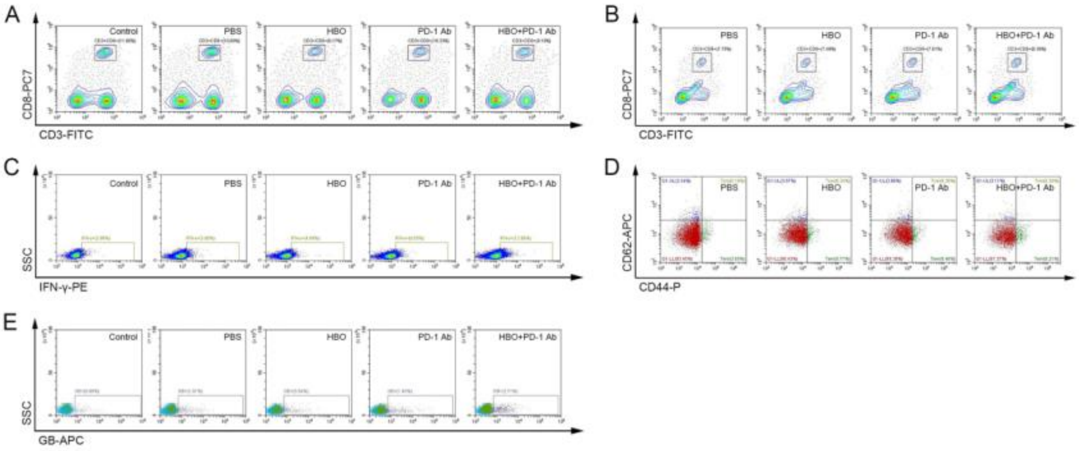

Figure S28. **Clustering diagram of flow cytometry analysis in relapse H22 tumor model.** Clustering diagram of CD8 (A), IFN-γ (C), granzyme B (E) of CTL, and CD8 (B), CD44/CD62 (D) of E, detected by flow cytometry in Fig.6.

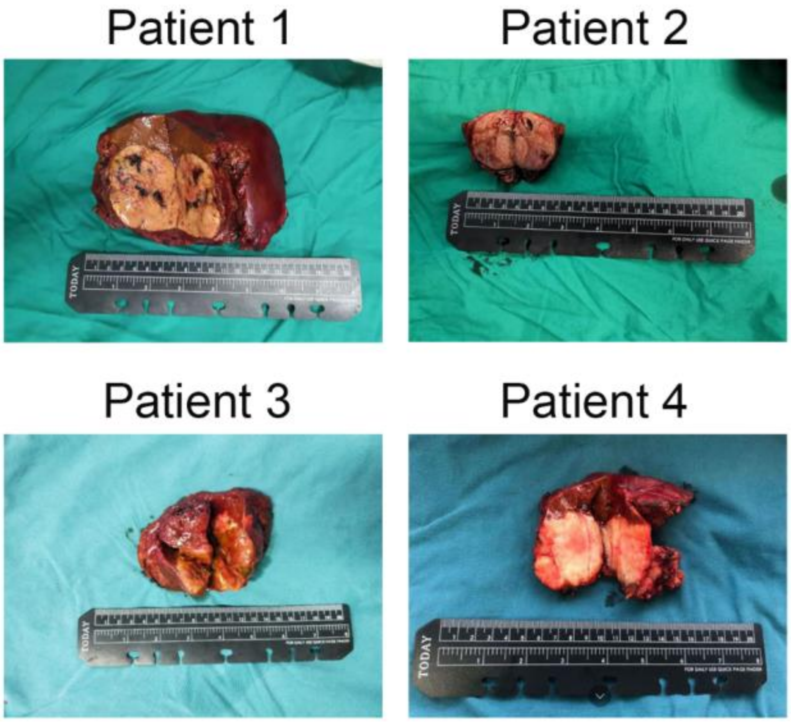

Figure S29. Clinical tumor sample images of different patients used in this study.

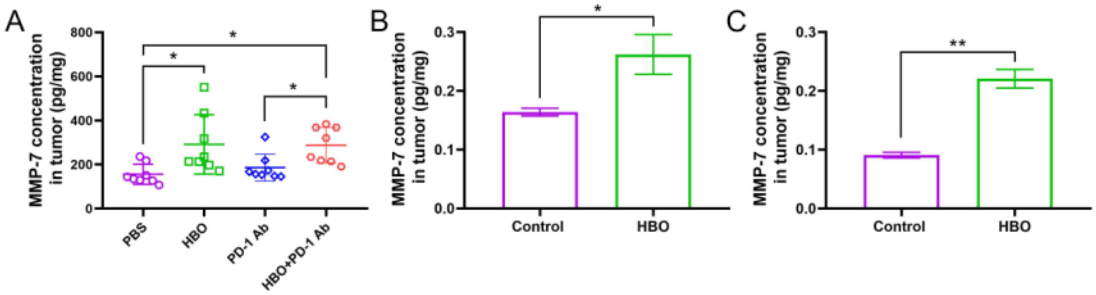

Figure S30. HBO increases MMP-7 expression in tumor tissues. (A) MMP-7 concentration in H22 orthotopic tumor after different treatments (n=8), (B, C) MMP-7 concentration of clinical tumor sample from two patients after treatments (n=3). Error bars indicate SEM. Statistical significance was calculated by t-test. P-values: *, $P < 0.05$; **, $P < 0.01$; ***, $P < 0.001$.

Table S1. Patient demographics.

Patient code	Age (years)	Sex	Tumor size (L x W, cm)
1	67	Male	7 x 6
2	70	Male	5 x 4
3	46	Male	7 x 6
4	69	Male	5.5 x 4.5
